# Genetic and environmental influences on sleep quality, ability to settle, and crying duration in 2‐ and 5‐month‐old infants: A longitudinal twin study

**DOI:** 10.1002/jcv2.70023

**Published:** 2025-07-04

**Authors:** Charlotte Viktorsson, Ashraf Yahia, Mark J. Taylor, Angelica Ronald, Kristiina Tammimies, Terje Falck‐Ytter

**Affiliations:** ^1^ Development and Neurodiversity Lab Department of Psychology Uppsala University Uppsala Sweden; ^2^ Division of Neuropsychiatry Department of Women's and Children's Health Center of Neurodevelopmental Disorders (KIND) Karolinska Institutet Stockholm Sweden; ^3^ Astrid Lindgren Children's Hospital Karolinska University Hospital Region Stockholm Sweden; ^4^ Department of Medical Epidemiology and Biostatistics Karolinska Institutet Stockholm Sweden; ^5^ School of Psychology Faculty of Health and Medical Sciences University of Surrey Guildford UK

**Keywords:** crying, infants, settle ability, sleep, twins

## Abstract

**Background:**

Sleep and behavioral regulation are both vital for early healthy development. Yet, little is known about the relative contribution of genetic and environmental factors to early sleep and regulatory behaviors, or how these etiological influences may change during the first months of life.

**Methods:**

Genetic and environmental influences on sleep, settle, and crying behaviors at 2 and 5 months were examined in 998 twins, using a classical twin design. In addition, polygenic scores were derived for a range of sleep behaviors, as well as psychiatric and neurodevelopmental conditions.

**Results:**

Genetic influences (A) explained a large part of the variation in duration of crying at both 2 and 5 months (A = 0.29–0.70) and in settle ability at 5 months (A = 0.51–0.67). Shared environment (C) primarily influenced number of wakeups per night at both ages (C = 0.61–0.90) and settle ability at 2 months (C = 0.36–0.65). Longitudinal analyses suggested modest shared genetic influence on settle ability in the daytime across the ages (24%), and non‐significant shared genetic estimates for ability to settle in the evening and at nighttime. There was moderate shared influence of shared environmental factors on number of wakeups per night (56%) and modest but significant shared genetic influence on crying duration in the evening and nighttime (17%–33%). Unique environmental effects were mostly specific to each age. Finally, autism polygenic score associated with longer crying duration in the evening at 2 months (*β* = 0.16, *p* = .002).

**Conclusions:**

Etiological influences tended to change from 2 to 5 months, reflecting a highly plastic period in infant brain development and in child‐environment interactions.

## Key Points

- Early sleep, settle, and crying behaviors in infants are important for various developmental outcomes.
- This study found that genetic factors largely influence crying duration and settle ability in infancy, while shared environmental factors primarily affect the number of night wakeups.
- Longitudinal analysis indicated modest shared genetic and environmental influences across 2–5 months of age.
- These findings underscore the importance of considering both genetic predispositions and environmental factors in infant care practices and parental guidance.

## Graphical abstract

This study found that genetic factors largely influence crying duration and settle ability in infancy, while shared environmental factors primarily affect the number of night wakeups. Etiological influences tended to change from 2 to 5 months, reflecting a highly plastic period in infant brain development and in child‐environment interactions.

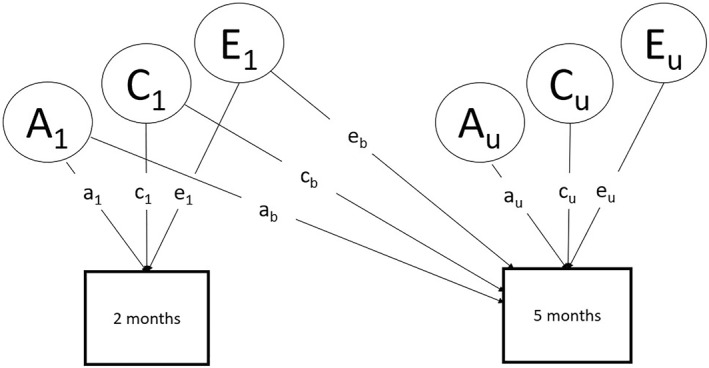

## INTRODUCTION

The autonomous regulation of sleep and wakefulness in infants marks a crucial developmental milestone in early childhood, reflecting alterations in neural activity across different regions of the brain (Kohyama, 1998). At around 4 months of age, a circadian rhythm based on a day‐night cycle develops (Heraghty et al., 2008), and infants gradually sleep less throughout the day, but increase their total sleep duration during the night (Figueiredo et al., 2016). While total 24‐h sleep duration and number of wakeups per night generally decreases throughout the first year of life (Galland et al., 2012), sleep quality is highly variable during this time and parent‐reported sleep problems are common (Paavonen et al., 2020). For some children, sleep disorders may persist into early childhood (Byars et al., 2012) and can be linked to behavioral and cognitive challenges (e.g., Dahl, 1996; Friedman et al., 2009; Gertner et al., 2002; Smaldone et al., 2007), making it an important target for potential interventions early in life. Because the progress of day‐ and nighttime sleep changes throughout development, it is necessary to study sleep at multiple time points in early childhood. Not only sleep behaviors are subject to developmental changes throughout the first year of life. One systematic review found mean fussing/crying durations of 117–133 min in the first 6 weeks, which dropped to 68 min at 10–12 weeks of age (Wolke et al., 2017). Similarly, another recent systematic review found mean fussing/crying durations of 78–126 min from 1 to 10 weeks of age, which dropped to 34–66 min from 11 to 38+ weeks (Vermillet et al., 2022). Excessive crying in infancy has an emotional impact on parents and their relationships, and parents often report a lack of support and a need for effective treatment (Muller et al., 2023). Information about the underlying etiology of fussing and crying in infancy may reduce stigma and might lead to new ways of supporting families.

A high heritability (64%) has been found for parent‐rated day/night sleep ratio at 6 months of age (Dionne et al., 2011), while studies of parent‐rated sleep duration and night awakenings in 15‐ to 18‐month‐olds have found a moderate heritability and a large influence of shared environmental factors (Brescianini et al., 2011; Fisher et al., 2012). One longitudinal study of parent‐rated sleep in infants at 6–48 months of age found varying but significant heritability of day‐ and nighttime sleep duration (15%–58%; Touchette et al., 2013). The pooled heritability estimates of sleep functions (including sleep duration, sleep problems, cortisol levels, etc.) in a meta‐analysis including approximately 6000 infants aged 0–2 years was 35% (Austerberry et al., 2022). However, none of the studies on sleep duration or sleep problems included in the meta‐analysis analyzed these behaviors in the first few months of life.

The main aim of this study was to estimate genetic and environmental contributions to different aspects of sleep and settle behaviors at 2 and 5 months of age, a time of significant developmental change. We chose to study these behaviors because they are important in relation to real‐life experiences of parents to young infants. Lack of sleep, excessive crying, or an unsettled child may be challenging for the family, and understanding the etiology behind these behaviors may inform future studies as well as practices and guidelines for parents. Based on earlier studies, we hypothesized that we would find significant genetic, shared environmental, and unique environmental influence on number of times the infant wakes up at night and the time it takes for the infant to settle. As crying and being easily upset has been found to be highly heritable at 2 years of age (Groen‐Blokhuis et al., 2011), we hypothesized that we would find some genetic influence on crying duration in early infancy, but we had no hypothesis regarding the magnitude of this influence. We also aimed to assess whether the genetic and environmental influences changed from 2 to 5 months. These ages were chosen due to the vast developmental changes happening in early infancy (which highlights the need to examine the etiology at multiple timepoints in the first years of life), and the lack of studies examining these behaviors at such young ages. While the twin design enables estimation of the net heritability of sleep behaviors, polygenic scores allow us to test whether common genetic variants associated with later outcomes (such as autism) associate with infant sleep behaviors. Polygenic scores for circadian preference have been associated with individual sleep trajectories (Merikanto et al., 2018), and polygenic scores for insomnia have been associated with sleep problems in childhood (Kocevska et al., 2024). It is unclear whether sleep‐related polygenic scores can predict sleep behaviors in early infancy, and therefore, we aimed to test the association between early sleep and settle behaviors and polygenic scores for circadian rhythm, insomnia, short sleep, and long sleep. As sleeping difficulties are prevalent in a range of neurodevelopmental and psychiatric conditions (Gregory & Sadeh, 2016), we additionally tested for associations between early sleep and settle behaviors and polygenic scores for common neurodevelopmental and psychiatric conditions. All hypotheses were preregistered (https://osf.io/g5jdp/).

## METHOD

### Participants

The sample consisted of 998 same‐sex Swedish twins, who are part of a longitudinal twin study called Gut‐2‐Twin. The participants were recruited via letters sent to families identified via the Swedish Total Population Register, targeting families who had a child in the right age range (1–2 months of age). Any exclusions were done later via parent interviews. In total, 1925 families were invited, and 510 decided to participate in the study. Gut‐2‐Twin did not include an on‐site visit and therefore recruited families throughout Sweden. Informed consent was obtained from all caregivers. The study was approved by the regional ethics board in Stockholm (2020‐03226) and was conducted in accordance with the Declaration of Helsinki.

Exclusion criteria for the Gut‐2‐Twin study were opposite‐sex twin pairs, diagnosis of epilepsy, known presence of genetic syndrome related to autism, uncorrected vision or hearing impairment, very premature birth (prior to week 34), presence of developmental or medical condition likely to affect brain development (e.g., Cerebral Palsy), birthweight below 1.5 kg, and infants without one of the biological parents involved in their care. Some participants were subsequently found not to fulfill the above criteria and were therefore excluded due to low birthweight, anomaly in magnetic resonance imaging or computed tomography scan, hearing impairment, cerebral palsy, seizures, and meningitis (*n* = 8 infants). In addition, nine infants were excluded due to lack of information about zygosity. For this study, we also excluded participants due to twin‐to‐twin transfusion syndrome (*n* = 17 pairs of twins). The children were rated by their parents on a number of abilities and difficulties at 2, 5, 14, 24, and 36 months of age. In addition, stool samples were collected (as part of a larger aim to study gut microbiota). Here, we used questionnaire data from the 2‐ and 5‐month assessments. As many twins are born prematurely, the age was corrected as chronological age minus the number of days between a full‐term pregnancy (39 weeks) and their gestational age. At both time points, using the corrected age, we excluded participants who were either 40 days younger or older than 2 months (60 days) and 5 months (150 days), respectively. Due to this criteria, 73 infants were excluded from the 2‐month assessment (due to being too old at time of assessment) and 34 infants were excluded from the 5‐month assessment (6 due to being too young, and 28 due to being too old). As a consequence, 11 participants were excluded from the study due to invalid age at both timepoints, and 15 were excluded due to invalid age at the only timepoint they provided any data. The final sample of participants that provided data for at least one time point was 921 infants. For 17 twin pairs, zygosity was determined based on questionnaire data (because the parents did not provide DNA samples or because the quality of the samples was too low), using a machine learning approach which has been found to have a prediction accuracy of 93.10%, with a sensitivity of 91.30% and specificity of 94.29% (see Hardiansyah et al., 2021 for details). For the rest of the sample, zygosity was determined by DNA analysis.

Data were collected on a number of background variables, such as birthweight, gestational age, family income, parental age, and approximate light exposure at the 2‐ and 5‐month assessments (due to large fluctuations in daylight in Sweden throughout the year). Daylight exposure was calculated based on the geographical location of the participant, consisting of the first number in the postal code (ranging from 1 to 9) of the family's home address. These postal codes were divided into four large areas covering Sweden, and daylight was calculated based on a city in the middle of each area, the 15th of each month. Daylight exposure (measured as minutes of daylight during a 24‐h period) was then calculated for each infant at each time of assessment, based on which month the parents filled in the questionnaire and which area of Sweden they resided in. While the pre‐registration stated that geographical location would be included as a background variable, it was subsequently excluded, as its primary use was the calculation of daylight exposure, and we did not expect geographical location in itself to influence the sleep, settle, and crying behaviors. Descriptive statistics of the demographic variables are presented in Table 1.

**TABLE 1:** Descriptive statistics of demographic variables.

	Mean (SD)^a^ [min; max]		
	Total (*n* = 921)	MZ (*n* = 484)	DZ (*n* = 437)
*N* females (%)	507 (55.0%)	278 (57.4%)	229 (52.4%)
Age^b^ at 2‐month assessment (days)	66.9 (15.4) [27; 99]	65.5 (16.2) [27; 98]	68.46 (14.5) [30; 99]
Age at 5‐month assessment (days)	142.3 (14.9) [110; 187]	139.9 (15.4) [110; 187]	145.0 (13.8) [111; 185]
Birthweight (gram)	2693.7 (387.3) [1630; 3950]	2595.0 (371.8) [1630; 3720]	2800.2 (375.7) [1736; 3950]
Gestational age (weeks)	36.69 (1.15) [34; 39]	36.40 (1.14) [34; 39]	37.01 (1.07) [34; 39]
Family income^c^	5.94 (2.22) [1; 10]	5.83 (2.05) [1; 10]	6.06 (2.40) [1; 10]
Maternal age (years)	31.86 (4.26) [20; 45]	31.36 (4.07) [20; 42]	32.40 (4.40) [22; 45]
Paternal age (years)	33.84 (5.52) [23; 59]	33.34 (4.68) [24; 47]	34.39 (6.25) [23; 59]
Daylight exposure at 2‐month assessment (minutes)	733.0 (245.1) [172; 1440]	744.6 (246.1) [172; 1440]	721.1 (244.0) [172; 1440]
Daylight exposure at 5‐month assessment (minutes)	780.2 (275.2) [172; 1440]	780.1 (276.4) [172; 1440]	780.3 (274.2) [172; 1257]

^a^ Except for *N* females, which shows the frequency.

^b^ Calculated as the difference between full term (39 weeks) and gestational age, subtracted from chronological age.

^c^ Family income per month. Scale 1–10 where 1 = <20K, 2 = 20–30K, 3 = 30–40K, 4 = 40–50K, 5 = 50–60K, 6 = 60–70K, 7 = 70–80K, 8 = 80–90K, 9 = 90–100K and 10 = >100K (SEK).

### Sleep, settle, and crying measures

The sleep, settle, and crying measures were derived from the Sleep and Settle Questionnaire (SSQ; Matthey, 2001) at 2 and 5 months of age. This is a 34‐item parent‐report questionnaire that assesses infant sleep and settling behavior. We extracted a subset of items: number of wakeups per night; time until settled during the day, evening, and night; and duration of crying during the day, evening, and night. These items were chosen as they cover important sleep and settle behaviors in early development (e.g., Brescianini et al., 2011; Wolke et al., 2017). At 2 months, the correlations among time until settled during the day, evening, and night ranged from 0.12 to 0.32, and the correlations among crying duration during the day, evening and night ranged from 0.30 to 0.54. At 5 months, the correlations among time until settled during the day, evening, and night ranged from 0.12 to 0.46, and the correlations among crying duration during the day, evening and night ranged from 0.35 to 0.53. We concluded that these correlations were not high enough to combine the measures into composite variables, and therefore analyzed the items separately. While the questionnaire also contained items on sleep duration, those questions were ambiguous and it was clear during preprocessing that caregivers interpreted those questions differently (i.e., the questions related to the duration of each “sleep period,” and while some parents reported total sleep duration, others reported duration of single sleep periods, making it difficult to discern the actual sleep duration of, e.g., the whole night, and to create a comparable variable). We, therefore, decided to exclude all items on sleep duration. The SSQ defines day as 5 a.m. to 6 p.m., evening as 6–10 p.m., and night as 10 p.m. to 5 a.m. All variables were reported as averages over the previous week. Durations were transformed to minutes and if parents reported a range (e.g., 10–30 min), the mean value was used. If a number less than zero was reported as the duration, without specifying the metric used, the answer was excluded (as it was unclear whether they replied in minutes or hours). Answers such as “a couple of minutes” or “a few minutes” were transformed to 2 min. While we also stated in the pre‐registered plan that we would extract the number of naps per day at each timepoint, we opted to exclude this variable considering the young age of participants.

### Statistical analyses

First, we estimated the association between each background variable and each sleep/settle measure at each timepoint. These associations were calculated using generalized estimating equations (GEE) in order to account for the correlation between twins in a pair (Carlin et al., 2005). If a background variable (such as family income) was associated with a sleep/settle measure, that background variable was regressed out of the sleep/settle measure before the twin analyses. Sex and age were regressed out from all variables before twin analyses. See Table S1 for a full overview of the GEE analyses of the background variables.

Univariate twin models were used to estimate the genetic and environmental contribution to each sleep/settle measure. The sources of variation in a trait can be divided into genetic influences (A; heritability), shared environment (C; environmental influences that makes twins similar to each other), and unique environment (E; i.e., environmental influences that makes twins different from each other, including measurement error). Since monozygotic (MZ) twins share 100% of their segregating DNA, while dizygotic (DZ) twins on average share 50% of their segregating DNA, a higher within pair similarity among MZ twins than DZ twins suggests genetic contribution to a trait. Where DZ twins are more than half as similar as MZ twins, this indicates the presence of C. The degree to which MZ twin similarity is lower than unity indicates the presence of E, which includes measurement error. In order to explore genetic and environmental influences, we calculated twin correlations and then fitted an ACE model (with AE, CE, and E nested models for comparison). A fully saturated model was first fitted in order to test the assumptions of equality of means and variances across zygosity and twin order. We found that variances were not equal across zygosity for some of the variables, and we therefore included a phenotypic interaction effect (denoted ‐s) in the ACE models, as per standard practice (Neale & Maes, 2004).

**FIGURE 1:**
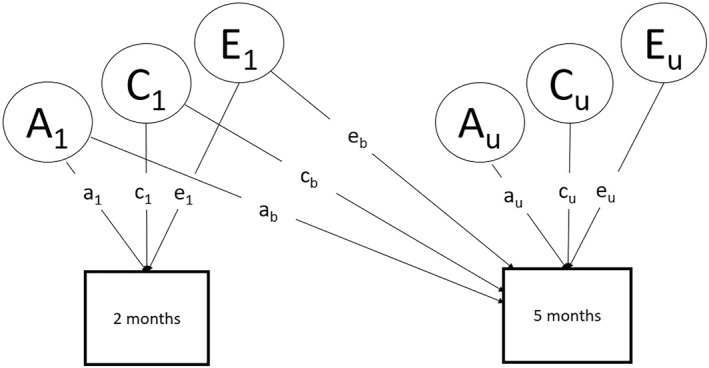
The longitudinal Cholesky decomposition. The latent variables (A, C, and E) represent the genetic, shared environmental, and unique environmental influences at each timepoint. Squaring the parameters a_1_, c_1_, and e_1_, and dividing them by the total variance, will give the proportion of variance in the sleep/settle measures at 2 months that are accounted for by the A_1_, C_1_, and E_1_ components. The parameters a_b_, c_b_, and e_b_ represent the carryover of genetic and environmental effects from 2 to 5 months. A_u_, C_u_, and E_u_ represent unique variance at 5 months, and squaring the parameters a_u_, c_u_, and e_u_, and dividing them by the total variance will give the proportion of variance at 5 months that is unique to that age.

We then calculated the phenotypic and cross‐trait cross‐twin correlations, and used a Cholesky decomposition to assess whether the genetic and environmental influences were unique or shared across ages (Figure 1). Model fit was decided based on the akaike information criterion value and non‐significance of the likelihood ratio test (i.e., no decrement in fit compared to the saturated or the genetic model, indexed by the *χ* ^2^ distribution).

Data analysis was performed in R 4.2.0 (R Core Team, 2017), and model fitting was performed through maximum likelihood optimization with OpenMx, version 2.20.6 (Neale et al., 2016).

### Polygenic scores

We derived polygenic scores based on genome‐wide association studies (GWAS's) for circadian rhythm (Jones et al., 2019), insomnia (Watanabe et al., 2022), short sleep (Austin‐Zimmerman et al., 2023), and long sleep (Austin‐Zimmerman et al., 2023). Because of the association between psychiatric and neurodevelopmental conditions and sleep, we also derived polygenic scores based on GWAS's for attention‐deficit/hyperactivity disorder (Demontis et al., 2023), autism (Grove et al., 2019), anxiety (Otowa et al., 2016), and depression (Howard et al., 2019). Full details of the genotyping and the calculations of polygenic scores are reported in Supporting Information S1, but a short summary is provided here.

Saliva samples were collected from the twins at home, and the DNA extraction was done at the KI‐biobank using the Hamilton ChemagicSTAR® platform. Quality control of the genotyping data was performed using PLINK 1.9 (Chang et al., 2015). Phasing and imputation were performed based on phase 3 of the 1000 genome project using the default parameters of SHAPEIT4 (Delaneau et al., 2019) and IMPUTE5 (Rubinacci et al., 2020), respectively. Polygenic scores were calculated using the default parameters of polygenic risk scores‐continuous shrinkage (Ge et al., 2019).

We then estimated the associations between early sleep and settle behaviors and polygenic scores separately for each phenotypic variable and each polygenic score, using GEE analyses. Both twins of each pair were included in the analysis, using cluster‐robust standard errors to account for relatedness. Sex, age, and any of the background variables that were significantly associated with the sleep and settle measures were included as covariates, along with the first 10 principal components of ancestry. We corrected for multiple testing using Bonferroni correction, separately for each polygenic score. As every score was used for 14 analyses (7 at each age point), the corrected *p*‐value was .004.

## RESULTS

Descriptive statistics of the sleep and settle measures at 2 and 5 months of age are presented in Table 2 (see Supporting Information S1 for distributional plots of all variables). Means were similar across zygosity and gender, but variations were large within each variable, reflecting the individual differences in sleep and settle behaviors at both 2 and 5 months of age.

**TABLE 2:** Descriptive statistics of sleep and settle measures.

	Mean (SD) [min; max]		
	Total	MZ twins	DZ twins
2‐month assessment			
Wakeups per night (*n*)	2.2 (1.1) [0; 10]	2.2 (1.1) [0; 10]	2.2 (1.1) [0; 7]
Time until settled (min)			
Daytime	15.0 (16.3) [0; 150]	15.3 (16.2) [0; 120]	14.7 (16.4) [0; 150]
Evening	26.7 (25.4) [0; 180]	26.8 (25.2) [0; 150]	26.5 (25.6) [0; 180]
Nighttime	18.2 (22.4) [0; 240]	18.5 (24.8) [0; 240]	17.9 (19.4) [0; 120]
Crying duration (min)			
Daytime	33.5 (39.6) [0; 360]	34.2 (40.6) [0; 300]	32.9 (38.5) [0; 360]
Evening	28.3 (32.7) [0; 240]	27.5 (32.1) [0; 240]	29.1 (33.4) [0; 180]
Nighttime	10.3 (17.9) [0; 180]	11.2 (19.6) [0; 180]	9.3 (15.8) [0; 120]
5‐month assessment			
Wakeups per night (*n*)	2.1 (1.4) [0; 10]	2.1 (1.4) [0; 9]	2.1 (1.5) [0; 10]
Time until settled (min)			
Daytime	11.2 (8.7) [0; 60]	10.9 (8.8) [0; 60]	11.5 (8.6) [0; 60]
Evening	17.8 (16.5) [0; 120]	18.3 (17.4) [0; 120]	17.3 (15.4) [0; 120]
Nighttime	12.7 (14.1) [0; 90]	12.9 (15.0) [0; 90]	12.5 (12.9) [0; 90]
Crying duration (min)			
Daytime	26.6 (33.6) [0; 300]	28.2 (36.9) [0; 300]	25.0 (29.6) [0; 240]
Evening	15.7 (18.6) [0; 120]	17.0 (20.2) [0; 120]	14.2 (16.5) [0; 120]
Nighttime	5.1 (9.6) [0; 120]	5.6 (10.1) [0; 120]	4.6 (9.0) [0; 90]

Twin correlations for each variable at both ages can be found in Table 3. Almost all twin correlations suggested genetic influence (i.e., an MZ correlation higher than the DZ correlation), except for time until settled at 2 months of age. Most correlations suggested influence from both genetic and shared environment (as the MZ correlation was higher, but less than twice as high, as the DZ correlation). Nonshared environment was implicated by the fact that the MZ correlations were all less than 1. We then checked the assumptions of twin modeling (equal means and variances across twins and zygosity) for each variable, details are reported in Table S2. As most variables showed a statistically significant difference in variance across zygosity, we included a phenotypic interaction effect in the twin analyses.

**TABLE 3:** Twin correlations for each sleep and settle measure at both ages, as well as phenotypic and cross‐twin cross‐trait correlations for all variables (across ages).

	Age 2‐month		Age 5‐month		Phenotypic	Cross‐twin cross‐trait	
	rMZ	rDZ	rMZ	rDZ		MZ	DZ
Wakeups per night	0.91 [0.89; 0.93]	0.76 [0.70; 0.81]	0.82 [0.78; 0.86]	0.54 [0.44; 0.62]	0.40 [0.31; 0.47]	0.38 [0.29; 0.46]	0.29 [0.20; 0.38]
Time until settled							
Daytime	0.76 [0.69; 0.81]	0.66 [0.57; 0.73]	0.67 [0.60; 0.73]	0.32 [0.19; 0.44]	0.29 [0.21; 0.37]	0.28 [0.19; 0.36]	0.22 [0.12; 0.31]
Evening	0.66 [0.57; 0.73]	0.51 [0.40; 0.61]	0.65 [0.58; 0.71]	0.44 [0.32; 0.55]	0.17 [0.08; 0.25]	0.12 [0.03; 0.21]	0.14 [0.03; 0.25]
Nighttime	0.65 [0.57; 0.71]	0.66 [0.56; 0.73]	0.64 [0.57; 0.70]	0.40 [0.27; 0.52]	0.28 [0.20; 0.36]	0.33 [0.24; 0.41]	0.34 [0.23; 0.44]
Crying duration							
Daytime	0.73 [0.66; 0.78]	0.59 [0.49; 0.68]	0.71 [0.64; 0.77]	0.56 [0.46; 0.65]	0.45 [0.38; 0.52]	0.34 [0.26; 0.42]	0.28 [0.18; 0.37]
Evening	0.78 [0.72; 0.83]	0.56 [0.44; 0.65]	0.78 [0.73; 0.82]	0.50 [0.38; 0.60]	0.30 [0.22; 0.38]	0.29 [0.21; 0.38]	0.21 [0.11; 0.31]
Nighttime	0.69 [0.58; 0.76]	0.56 [0.42; 0.66]	0.64 [0.56; 0.71]	0.34 [0.20; 0.46]	0.38 [0.29; 0.46]	0.30 [0.19; 0.41]	0.27 [0.15; 0.38]

*Note*: 95% confidence intervals shown in brackets.

Univariate twin models were then fitted to all variables at both timepoints. For brevity, only the selected model for each variable are shown in Table 4, but full results are reported in Tables S3–S16.

**TABLE 4:** Summary of model fitting for the selected univariate twin models.

	Model	−2LL	AIC	Comparison model	Δ*χ* ^2^	*p*	A	C	E	s
2 months										
Wakeups per night	ACE	1553.30	1561.30	ACE‐s	0.02	.88	0.30 [0.20; 0.43]	0.61 [0.48; 0.71]	0.09 [0.07; 0.12]	‐
Settle (daytime)	ACE	1739.37	1747.37	ACE‐s	0.02	.88	0.19 [0.02; 0.38]	0.56 [0.39; 0.71]	0.25 [0.20; 0.31]	‐
Settle (evening)	ACE	1740.89	1748.89	ACE‐s	0.13	.72	0.29 [0.05; 0.55]	0.36 [0.13; 0.56]	0.34 [0.27; 0.43]	‐
Settle (nighttime)	CE	1789.82	1795.82	ACE‐s	1.54	.46	‐	0.65 [0.58; 0.70]	0.35 [0.30; 0.42]	‐
Crying (daytime)	AE‐s	1785.42	1793.42	ACE‐s	<0.001	1.00	0.48 [0.18; 0.66]	‐	0.52 [0.34; 0.82]	0.17 [0.09; 0.25]
Crying (evening)	ACE	1692.04	1700.04	ACE‐s	0.01	.91	0.44 [0.24; 0.67]	0.34 [0.12; 0.52]	0.22 [0.17; 0.28]	‐
Crying (nighttime)	AE‐s	1874.29	1882.29	ACE‐s	<0.001	1.00	0.46 [0.20; 0.64]	‐	0.54 [0.36; 0.80]	0.16 [0.08; 0.23]
5 months										
Wakeups per night	ACE	2170.45	2178.45	ACE‐s	3.72	.05	0.56 [0.40; 0.76]	0.26 [0.07; 0.42]	0.18 [0.14; 0.22]	‐
Settle (daytime)	AE	2202.74	2208.74	ACE‐s	0.01	.99	0.67 [0.59; 0.73]	‐	0.33 [0.27; 0.41]	‐
Settle (evening)	AE‐s	2280.64	2288.64	ACE‐s	<0.001	1.00	0.52 [0.30; 0.67]	‐	0.48 [0.33; 0.70]	0.10 [0.01; 0.17]
Settle (nighttime)	AE‐s	2279.50	2288.64	ACE‐s	<0.001	1.00	0.51 [0.29; 0.66]	‐	0.49 [0.34; 0.71]	0.09 [0.001; 0.17]
Crying (daytime)	AE‐s	2212.23	2220.23	ACE‐s	<0.001	1.00	0.29 [0.02; 0.50]	‐	0.71 [0.50; 0.98]	0.21 [0.14; 0.27]
Crying (evening)	AE‐s	2149.05	2157.05	ACE‐s	<0.001	1.00	0.70 [0.56; 0.78]	‐	0.30 [0.22; 0.44]	0.09 [0.02; 0.16]
Crying (nighttime)	AE	2087.97	2093.97	ACE‐s	0.34	.84	0.64 [0.56; 0.71]	‐	0.36 [0.29; 0.44]	‐

*Note*: −2LL: minus 2 log likelihood fit statistic. AIC: akaike information criterion, lower value denotes better model fit. Δ*χ* ^2^: Change in −2LL statistic between two models, distributed as *χ* ^2^. 95% confidence intervals shown in brackets.

In summary, shared environment had a moderate‐to‐large influence on all measures at 2 months of age, except for crying duration at day‐ and nighttime. However, at 5 months of age, only number of wakeups per night showed an influence of shared environment (which accounted for 90% of variance in wakeups). At 2 months, genetic influence was modest‐to‐moderate for all variables. At 5 months, all variables except for wakeups per night and crying duration during daytime showed a larger heritability estimate, suggesting that the heritability of these behaviors increases over time (although it is noted that most confidence intervals overlapped between age 2‐ and 5‐month heritability estimates). When only age and sex covariates were regressed out, as requested by a reviewer, conclusions did not change (see Supporting Information S1 for those results).

In order to assess the amount of shared variance across ages, we calculated the phenotypic and cross‐twin cross‐trait correlations for all variables (see Table 3), and then fitted bivariate Cholesky decompositions to all variables (across ages). For brevity, only the selected model for each variable is shown in Table 5, but full results are reported in Tables S17–S23. A modest but significant proportion of the genetic influence on crying duration in the evening and nighttime at 5 months was genetic variance shared with the 2‐month timepoint (17%–33%). This was true also for settle ability during the day (shared genetic variance = 24%), but the shared genetic estimates for settle ability at other times of the day were non‐significant. The majority of the shared environmental influence on wakeups per night at 5 months was environmental variance shared with the 2‐month timepoint (56%), while a modest but significant proportion of the genetic influence was shared with 2 months (16%). For crying duration during daytime, 15% of the unique environmental influence was shared across timepoints, but no other significant shared influences were found for unique environmental factors.

**TABLE 5:** Summary of model fitting for the selected bivariate twin models.

	Model	−2LL	AIC	Comparison model	Δ*χ* ^2^	*p*	A	C	E	Shared A	Shared C	Shared E	Unique A	Unique C	Unique E
							2 months	2 months	2 months						
Wakeups per night	ACE	3651.11	3673.11	ACE‐s	1.90	.39	0.30 [0.20; 0.43]	0.61 [0.48; 0.71]	0.09 [0.07; 0.12]	0.03 [0.004; 0.09]	0.42 [0.32; 0.53]	<0.001 [<0.001; 0.003]	0.16 [0.09; 0.23]	0.33 [0.21; 0.45]	0.05 [0.04; 0.07]
Time until settled (daytime)	AE‐s	3899.98	3919.98	ACE‐s	1.87	.60	0.42 [0.07; 0.63]	‐	0.58 [0.37; 0.93]	0.16 [0.07; 0.68]	‐	<0.001 [<0.001; 0.007]	0.51 [<0.001; 0.64]	‐	0.33 [0.24; 0.48]
Time until settled (evening)	AE‐s	4008.06	4028.06	ACE‐s	2.75	.43	0.45 [0.05; 0.65]	‐	0.55 [0.35; 0.95]	0.02 [<0.001; 0.20]	‐	0.005 [<0.001; 0.03]	0.50 [0.28; 0.65]	‐	0.47 [0.33; 0.69]
Time until settled (nighttime)	ACE	4013.49	4035.49	ACE‐s	1.11	.57	<0.001 [<0.001; 0.19]	0.65 [0.48; 0.71]	0.35 [0.29; 0.41]	0.46 [<0.001; 0.60]	0.18 [0.05; 0.33]	0.007 [<0.001; 0.03]	<0.001 [<0.001; 0.56]	<0.001 [<0.001; 0.26]	0.35 [0.29; 0.43]
Crying duration (daytime)	ACE	3855.74	3877.74	ACE‐s	4.68	.10	0.12 [0.04; 0.21]	0.79 [0.70; 0.85]	0.10 [0.08; 0.12]	0.02 [<0.001; 0.07]	0.41 [29; 0.55]	0.02 [0.01; 0.03]	<0.001 [<0.001; 0.05]	0.45 [0.28; 0.58]	0.11 [0.09; 0.13]
Crying duration (evening)	AE‐s	3792.43	3812.43	ACE‐s	0.22	.97	0.67 [0.49; 0.77]	‐	0.33 [0.23; 0.51]	0.12 [0.06; 0.21]	‐	<0.001 [<0.001; 0.01]	0.58 [0.44; 0.69]	‐	0.30 [0.22; 0.43]
Crying duration (nighttime)	AE‐s	3903.67	3923.67	ACE‐s	<0.001	1.00	0.46 [0.18; 0.64]	‐	0.54 [0.36; 0.82]	0.20 [0.08; 0.47]	‐	0.004 [<0.001; 0.04]	0.41 [0.13; 0.58]	‐	0.39 [0.27; 0.57]

*Note*: −2LL: minus 2 log likelihood fit statistic. AIC: akaike information criterion, lower value denotes better model fit. Δ*χ* ^2^: change in −2LL statistic between two models, distributed as *χ* ^2^. 95% confidence intervals shown in brackets.

A statistically significant positive association was found between polygenic score for autism and crying duration in the evening at 2 months (*β* = 0.16, *p* = .002, *R* ^2^ = 0.02), meaning that a higher polygenic score was related to longer periods of crying. No other significant associations were found between the polygenic scores and sleep/settle measures at any timepoint (see Table S24 for details).

## DISCUSSION

Previous research has found that sleep quality in early childhood is linked to numerous developmental outcomes (e.g., Friedman et al., 2009; Gertner et al., 2002; Smaldone et al., 2007), and understanding the nature of early sleep and settle behaviors is an important step in elucidating the link between early sleep and later development. The aim of this study was to assess the genetic and environmental influence on a range of sleep and settle behaviors at 2 and 5 months of age, and to assess whether the familial and environmental influences were shared across these timepoints. In short, shared environmental factors influenced individual differences in number of wakeups per night at both ages, and settle abilities at 2 months. At 5 months, settle ability was primarily influenced by genetics. Duration of crying at different times of the day showed a moderate‐to‐high heritability at both 2 and 5 months. In general, the shared genetic influence from 2 to 5 months of age was low, while significant unique genetic effects were present at 5 months.

A large influence from shared environment was found for number of wakeups per night at 2 and 5 months, and time until settled during the night at 2 months, suggesting that shared environmental factors are especially important during nighttime. The majority of the shared environmental influence at 5 months was influence shared with the 2‐month assessment (56%), suggesting that some environmental factors are stable across these timepoints and significantly impact night sleep in infants. While we controlled for a range of background variables, there are several other factors that may influence night awakenings and the time it takes for the infant to settle once they have woken up. For example, infants who wake up frequently during the night are more likely to be breastfed and to have mothers with depression than infants who sleep through the night (Weinraub et al., 2012). It has also been found that infants whose crib was located in the parents' bedroom were more likely to not be self‐soothing during the night, as compared to infants who did not sleep in the same room as their parents (Goodlin‐Jones et al., 2001). In this study, we aimed to explore the etiology behind early sleep and settle behaviors, and by doing so, lay the groundwork for future studies on specific genetic and environmental aspects that may influence these behaviors. Thus, while we did not measure parental practices for sleeping and soothing, or parental depressive symptomatology, in this study, future research should focus on finding environmental predictors of night awakenings and settle ability, which may be important targets for future sleep interventions.

A moderate‐to‐large influence of genetics was found for crying duration at all times of the day at both 2 and 5 months, which is in line with an earlier study finding high heritability of crying and being easily upset at 2 years of age (Groen‐Blokhuis et al., 2011). The longitudinal analysis suggested a shared genetic influence between 2 and 5 months that was modest for evening and nighttime crying (17%–33%). At 5 months, there were large and significant unique genetic effects, meaning that novel genetic influence come into play at this timepoint.

For several variables, variances differed across zygosity, and we therefore included a sibling interaction term in the analyses (in accordance with general practice; see Neale & Maes, 2004). A positive sibling interaction effect was found for crying duration at day‐ and nighttime at 2 months, and for ability to settle in the evening and night, as well as crying duration at daytime and evening at 5 months of age. This means that the behavior in one twin leads to similar behavior in their co‐twin. This might reflect a true sibling interaction effect, whereby, for example, the crying of one twin induces crying in the other twin, leading to a prolonged duration of crying for both twins and a longer time until they settle down. However, these interaction effects may also reflect rater bias. When parents rate their children's behavior, they may (consciously or unconsciously) use one twin as a standard against which the behavior of the co‐twin is rated. By doing so, the parents may exacerbate either the similarities or differences between the children, resulting in what seems like interaction effects. For example, in this study it may manifest as a tendency to report a longer duration of crying in the co‐twin if the first twin cries for extended periods of time. By modeling the sibling interaction effect, we can avoid falsely attributing variance to shared environment, making the estimates we get from the ACE model more accurate. Due to the finding of sibling interaction effects, and because caring for more than one infant at once poses higher demands for the parents, the generalizability of the findings from an infant twin study of sleep behaviors might be questionable. However, in a comparison study using the 5‐month assessment of this sample and a sample of singletons of the same age, no differences between twins and singletons were found regarding settle and crying behaviors (Viktorsson et al., 2024). There was a statistically significant difference in the number of wakeups, but the twins woke up *less* frequently than the singletons, and the number of wakeups in the twin sample was similar to what has been found in other studies of singletons (e.g., Paavonen et al., 2020). Therefore, despite the unique demands of twin parents, it is reasonable to believe that these findings are generalizable to singletons.

While the sample in this study was typically developing, some of the parents reported very extended durations of crying (Table 2). Excessive crying in infancy is sometimes referred to as colic (e.g., Wolke et al., 2002) and has been associated with behavioral problems, hyperactivity, and mood problems at 4–6 years of age (Santos et al., 2015; Smarius et al., 2017), and hyperactivity problems and academic difficulties at 8–10 years of age (Wolke et al., 2002). Here, we found a statistically significant association between polygenic score for autism and crying duration in the evening at 2 months of age, meaning that a higher genetic predisposition for autism was related to longer periods of crying. An earlier study found that parents retrospectively reported a significantly higher rate of persistent crying in infants with later autism than in typically developing infants (Bag et al., 2018), and already at 1 month of age the cries of infants with later autism has been rated as less typical than the cries of children without later diagnosis (English et al., 2019). However, we did not find an association between autism polygenic score and crying duration at any other time of the day, or at 5 months of age, meaning that the potential link between genetic predisposition for autism and infant crying should be examined further and that the above‐mentioned finding should be interpreted cautiously. No other associations were found between polygenic scores and sleep/settle behaviors. This may reflect a lack of predictive power for these polygenic scores on early infant behavior, but it may also be the case that the sleep polygenic scores assessed in adult populations may not reflect the genetic underpinnings of sleep behaviors in early infancy.

It is important to note that we relied on parent‐reported questionnaires, and it is possible that the values reported by the parents might not reflect the sleep and settle behaviors accurately. While one study of 3–4‐year‐olds found that the correlation between parent‐reports and objectively measured sleep duration was high (Sekine et al., 2002), others have found some discrepancies between actigraphy‐obtained and mother‐reported sleep durations (e.g., Simard et al., 2013). Future studies should focus on minimizing the risk for different interpretations of questions, either by clarifications in the questionnaire or by obtaining the answers by conducting an interview with the parents.

## CONCLUSIONS

In a sample of more than 900 infant twins, we found that shared environmental factors influenced individual differences in number of wakeups per night at 2 and 5 months of age, and settle abilities at 2 months. Duration of crying showed a moderate‐to‐high heritability at both 2 and 5 months. Shared genetic influence across time was low for all measures, while significant unique genetic effects were present at 5 months. Unique environmental effects were mostly specific to each time point. Autism polygenic score associated with longer crying duration in the evening at 2 months, but no other associations with polygenic scores were found. In summary, this study sheds new light on the etiological factors underlying sleep, settle, and crying behaviors in early infancy, suggesting significant changes in etiology during the first months of life.

## AUTHOR CONTRIBUTIONS

**Charlotte Viktorsson**: Conceptualization; data curation; formal analysis; writing—original draft. **Ashraf Yahia**: Data curation; writing—review and editing. **Mark J. Taylor**: Conceptualization; methodology; software; writing—review and editing. **Angelica Ronald**: Conceptualization; funding acquisition; methodology; writing—review and editing. **Kristiina Tammimies**: Conceptualization; funding acquisition; methodology; writing—review and editing. **Terje Falck‐Ytter**: Conceptualization; funding acquisition; project administration; resources; supervision; writing—review and editing.

## CONFLICT OF INTEREST STATEMENT

The authors declare no conflicts of interest.

## ETHICAL CONSIDERATIONS

Informed consent was obtained from all caregivers. The study was approved by the regional ethics board in Stockholm (2020‐03226) and was conducted in accordance with the Declaration of Helsinki.

## Supporting information

Supporting Information S1

## Data Availability

The analyses presented here were preregistered (https://osf.io/g5jdp/). The data and code necessary to reproduce the analyses presented here are not publicly accessible, but will be made available upon reasonable request to the corresponding author. Note that sharing of pseudonymized personal data will require a data sharing agreement, according to Swedish and EU law.

## References

[jcv270023-bib-0001] Austerberry, C. , Mateen, M. , Fearon, P. , & Ronald, A. (2022). Heritability of psychological traits and developmental milestones in infancy: A systematic review and meta‐analysis. JAMA Network Open, 5(8), e2227887. 10.1001/jamanetworkopen.2022.27887 35994288 PMC9396365

[jcv270023-bib-0002] Austin‐Zimmerman, I. , Levey, D. F. , Giannakopoulou, O. , Deak, J. D. , Galimberti, M. , Adhikari, K. , Zhou, H. , Denaxas, S. , Irizar, H. , Kuchenbaecker, K. , McQuillin, A. , Concato, J. , Buysse, D. J. , Gaziano, J. M. , Gottlieb, D. J. , Polimanti, R. , Stein, M. B. , Bramon, E. , & Gelernter, J. (2023). Genome‐wide association studies and cross‐population meta‐analyses investigating short and long sleep duration. Nature Communications, 14(1), 1–15. 10.1038/s41467-023-41249-y PMC10539313 37770476

[jcv270023-bib-0003] Bag, O. , Alsen Guney, S. , Cevher Binici, N. , Tuncel, T. , Sahin, A. , Berksoy, E. , & Ecevit, C. (2018). Infant colic or early symptom of autism spectrum disorder? Pediatrics International, 60(6), 517–522. 10.1111/ped.13565 29573066

[jcv270023-bib-0004] Brescianini, S. , Volzone, A. , Fagnani, C. , Patriarca, V. , Grimaldi, V. , Lanni, R. , Serino, L. , Mastroiacovo, P. , Antonietta Stazi, M. , & Stazi, M. A. (2011). Genetic and environmental factors shape infant sleep patterns: A study of 18‐month‐old twins. Pediatrics, 127(5), e1296–e1302. 10.1542/peds.2010-0858 21482604

[jcv270023-bib-0005] Byars, K. C. , Yolton, K. , Rausch, J. , Lanphear, B. , & Beebe, D. W. (2012). Prevalence, patterns, and persistence of sleep problems in the first 3 years of life. Pediatrics, 129(2), e276–e284. 10.1542/peds.2011-0372 22218837 PMC3357046

[jcv270023-bib-0006] Carlin, J. B. , Gurrin, L. C. , Sterne, J. A. C. , Morley, R. , & Dwyer, T. (2005). Regression models for twin studies: A critical review. International Journal of Epidemiology, 34(5), 1089–1099. 10.1093/ije/dyi153 16087687

[jcv270023-bib-0007] Chang, C. C. , Chow, C. C. , Tellier, L. C. , Vattikuti, S. , Purcell, S. M. , & Lee, J. J. (2015). Second‐generation PLINK: Rising to the challenge of larger and richer datasets. GigaScience, 4(1), 7. 10.1186/s13742-015-0047-8 25722852 PMC4342193

[jcv270023-bib-0008] Dahl, R. E. (1996). The impact of inadequate sleep on children's daytime cognitive function. Seminars in Pediatric Neurology, 3(1), 44–50. 10.1016/s1071-9091(96)80028-3 8795841

[jcv270023-bib-0009] Delaneau, O. , Zagury, J. F. , Robinson, M. R. , Marchini, J. L. , & Dermitzakis, E. T. (2019). Accurate, scalable and integrative haplotype estimation. Nature Communications, 10(1), 5436. 10.1038/s41467-019-13225-y PMC6882857 31780650

[jcv270023-bib-0010] Demontis, D. , Walters, G. B. , Athanasiadis, G. , Walters, R. , Therrien, K. , Nielsen, T. T. , Farajzadeh, L. , Voloudakis, G. , Bendl, J. , Zeng, B. , Zhang, W. , Grove, J. , Als, T. D. , Duan, J. , Satterstrom, F. K. , Bybjerg‐Grauholm, J. , Bækved‐Hansen, M. , Gudmundsson, O. O. , Magnusson, S. H. , … Børglum, A. D. (2023). Genome‐wide analyses of ADHD identify 27 risk loci, refine the genetic architecture and implicate several cognitive domains. Nature Genetics, 55(2), 198–208. 10.1038/s41588-022-01285-8 36702997 PMC10914347

[jcv270023-bib-0011] Dionne, G. , Touchette, E. , Forget‐Dubois, N. , Petit, D. , Tremblay, R. E. , Montplaisir, J. Y. , & Boivin, M. (2011). Associations between sleep‐wake consolidation and language development in early childhood: A longitudinal twin study. Sleep, 34(8), 987–995. 10.5665/SLEEP.1148 21804661 PMC3138173

[jcv270023-bib-0012] English, M. S. , Tenenbaum, E. J. , Levine, T. P. , Lester, B. M. , & Sheinkopf, S. J. (2019). Perception of cry characteristics in 1‐month‐old infants later diagnosed with autism spectrum disorder. Journal of Autism and Developmental Disorders, 49(3), 834–844. 10.1007/s10803-018-3788-2 30361941 PMC10266897

[jcv270023-bib-0013] Figueiredo, B. , Dias, C. C. , Pinto, T. M. , & Field, T. (2016). Infant sleep‐wake behaviors at two weeks, three and six months. Infant Behavior and Development, 44, 169–178. 10.1016/j.infbeh.2016.06.011 27448323

[jcv270023-bib-0014] Fisher, A. , van Jaarsveld, C. H. , Llewellyn, C. H. , & Wardle, J. (2012). Genetic and environmental influences on infant sleep. Pediatrics, 129(6), 1091–1096. 10.1542/peds.2011-1571 22585775

[jcv270023-bib-0015] Friedman, N. P. , Corley, R. P. , Hewitt, J. K. , & Wright, K. P., Jr. (2009). Individual differences in childhood sleep problems predict later cognitive executive control. Sleep, 32(3), 323–333. 10.1093/sleep/32.3.323 19294952 PMC2647786

[jcv270023-bib-0016] Galland, B. C. , Taylor, B. J. , Elder, D. E. , & Herbison, P. (2012). Normal sleep patterns in infants and children: A systematic review of observational studies. Sleep Medicine Reviews, 16(3), 213–222. 10.1016/j.smrv.2011.06.001 21784676

[jcv270023-bib-0017] Ge, T. , Chen, C. Y. , Ni, Y. , Feng, Y. C. A. , & Smoller, J. W. (2019). Polygenic prediction via Bayesian regression and continuous shrinkage priors. Nature Communications, 10(1), 1776. 10.1038/s41467-019-09718-5 PMC6467998 30992449

[jcv270023-bib-0018] Gertner, S. , Greenbaum, C. W. , Sadeh, A. , Dolfin, Z. , Sirota, L. , & Ben‐Nun, Y. (2002). Sleep‐wake patterns in preterm infants and 6 month's home environment: Implications for early cognitive development. Early Human Development, 68(2), 93–102. 10.1016/s0378-3782(02)00018-x 12113995

[jcv270023-bib-0019] Goodlin‐Jones, B. L. , Burnham, M. M. , Gaylor, E. E. , & Anders, T. F. (2001). Night waking, sleep‐wake organization, and self‐soothing in the first year of life. Journal of Developmental and Behavioral Pediatrics, 22(4), 226–233. 10.1097/00004703-200108000-00003 11530895 PMC1201414

[jcv270023-bib-0020] Gregory, A. M. , & Sadeh, A. (2016). Annual Research Review: Sleep problems in childhood psychiatric disorders‐‐A review of the latest science. Journal of Child Psychology and Psychiatry, 57(3), 296–317. 10.1111/jcpp.12469 26412255

[jcv270023-bib-0021] Groen‐Blokhuis, M. M. , Middeldorp, C. M. , van Beijsterveldt, C. E. M. , & Boomsma, D. I. (2011). Crying without a cause and being easily upset in two‐year‐olds: Heritability and predictive power of behavioral problems. Twin Research and Human Genetics, 14(5), 393–400. 10.1375/twin.14.5.393 21962130

[jcv270023-bib-0022] Grove, J. , Ripke, S. , Als, T. D. , Mattheisen, M. , Walters, R. K. , Won, H. , Pallesen, J. , Agerbo, E. , Andreassen, O. A. , Anney, R. , Awashti, S. , Belliveau, R. , Bettella, F. , Buxbaum, J. D. , Bybjerg‐Grauholm, J. , Bækvad‐Hansen, M. , Cerrato, F. , Chambert, K. , Christensen, J. H. , … Børglum, A. D. (2019). Identification of common genetic risk variants for autism spectrum disorder. Nature Genetics, 51(3), 431–444. 10.1038/S41588-019-0344-8 30804558 PMC6454898

[jcv270023-bib-0023] Hardiansyah, I. , Hamrefors, L. , Siqueiros, M. , Falck‐Ytter, T. , & Tammimies, K. (2021). Determining zygosity in infant twins ‐ Revisiting the questionnaire approach. Twin Research and Human Genetics, 24(3), 168–175. 10.1017/thg.2021.24 34247691

[jcv270023-bib-0024] Heraghty, J. L. , Hilliard, T. N. , Henderson, A. J. , & Fleming, P. J. (2008). The physiology of sleep in infants. Archives of Disease in Childhood, 93(11), 982–985. 10.1136/adc.2006.113290 18653626

[jcv270023-bib-0025] Howard, D. M. , Adams, M. J. , Clarke, T. K. , Hafferty, J. D. , Gibson, J. , Shirali, M. , Coleman, J. R. I. , Hagenaars, S. P. , Ward, J. , Wigmore, E. M. , Alloza, C. , Shen, X. , Barbu, M. C. , Xu, E. Y. , Whalley, H. C. , Marioni, R. E. , Porteous, D. J. , Davies, G. , Deary, I. J. , … McIntosh, A. M. (2019). Genome‐wide meta‐analysis of depression identifies 102 independent variants and highlights the importance of the prefrontal brain regions. Nature Neuroscience, 22(3), 343–352. 10.1038/s41593-018-0326-7 30718901 PMC6522363

[jcv270023-bib-0026] Jones, S. E. , Lane, J. M. , Wood, A. R. , van Hees, V. T. , Tyrrell, J. , Beaumont, R. N. , Jeffries, A. R. , Dashti, H. S. , Hillsdon, M. , Ruth, K. S. , Tuke, M. A. , Yaghootkar, H. , Sharp, S. A. , Jie, Y. , Thompson, W. D. , Harrison, J. W. , Dawes, A. , Byrne, E. M. , Tiemeier, H. , … Weedon, M. N. (2019). Genome‐wide association analyses of chronotype in 697,828 individuals provides insights into circadian rhythms. Nature Communications, 10(1), 1–11. 10.1038/s41467-018-08259-7 PMC6351539 30696823

[jcv270023-bib-0027] Kocevska, D. , Trajanoska, K. , Mulder, R. H. , Koopman‐Verhoeff, M. E. , Luik, A. I. , Tiemeier, H. , & van Someren, E. J. W. (2024). Are some children genetically predisposed to poor sleep? A polygenic risk study in the general population. Journal of Child Psychology and Psychiatry, 65(5), 710–719. 10.1111/jcpp.13899 37936537

[jcv270023-bib-0028] Kohyama, J. (1998). Sleep as a window on the developing brain. Current Problems in Pediatrics, 28(3), 69–92. 10.1016/s0045-9380(98)80054-6 9571325

[jcv270023-bib-0029] Matthey, S. (2001). The sleep and settle questionnaire for parents of infants: Psychometric properties. Journal of Paediatrics and Child Health, 37(5), 470–475. 10.1046/j.1440-1754.2001.00703.x 11885711

[jcv270023-bib-0030] Merikanto, I. , Lahti, J. , Kuula, L. , Heinonen, K. , Raikkonen, K. , Andersson, S. , Strandberg, T. , & Pesonen, A. K. (2018). Circadian preference and sleep timing from childhood to adolescence in relation to genetic variants from a genome‐wide association study. Sleep Medicine, 50, 36–41. 10.1016/j.sleep.2018.04.015 29982088

[jcv270023-bib-0031] Muller, I. , Ghio, D. , Mobey, J. , Jones, H. , Hornsey, S. , Dobson, A. , Maund, E. , & Santer, M. (2023). Parental perceptions and experiences of infant crying: A systematic review and synthesis of qualitative research. Journal of Advanced Nursing, 79(2), 403–417. 10.1111/jan.15492 36373818 PMC10100257

[jcv270023-bib-0032] Neale, M. C. , Hunter, M. D. , Pritikin, J. N. , Zahery, M. , Brick, T. R. , Kirkpatrick, R. M. , Estabrook, R. , Bates, T. C. , Maes, H. H. , & Boker, S. M. (2016). OpenMx 2.0: Extended structural equation and statistical modeling. Psychometrika, 81(2), 535–549. 10.1007/s11336-014-9435-8 25622929 PMC4516707

[jcv270023-bib-0033] Neale, M. C. , & Maes, H. H. (2004). Methodology for genetic studies of twins and families. Kluwer Academics.

[jcv270023-bib-0034] Otowa, T. , Hek, K. , Lee, M. , Byrne, E. M. , Mirza, S. S. , Nivard, M. G. , Bigdeli, T. , Aggen, S. H. , Adkins, D. , Wolen, A. , Fanous, A. , Keller, M. C. , Castelao, E. , Kutalik, Z. , Der Auwera, S. V. , Homuth, G. , Nauck, M. , Teumer, A. , Milaneschi, Y. , … Hettema, J. M. (2016). Meta‐analysis of genome‐wide association studies of anxiety disorders. Molecular Psychiatry, 21(10), 1391–1399. 10.1038/mp.2015.197 26754954 PMC4940340

[jcv270023-bib-0035] Paavonen, E. J. , Saarenpaa‐Heikkila, O. , Morales‐Munoz, I. , Virta, M. , Hakala, N. , Polkki, P. , Kylliäinen, A. , Karlsson, H. , Paunio, T. , & Karlsson, L. (2020). Normal sleep development in infants: Findings from two large birth cohorts. Sleep Medicine, 69, 145–154. 10.1016/j.sleep.2020.01.009 32087408

[jcv270023-bib-0036] R Core Team . (2017). R: A language and environment for statistical computing [Computer software manual]. R Foundation for Statistical Computing. Retrieved from. http://www.R‐project.org/

[jcv270023-bib-0037] Rubinacci, S. , Delaneau, O. , & Marchini, J. (2020). Genotype imputation using the positional burrows wheeler transform. PLoS Genetics, 16(11), e1009049. 10.1371/journal.pgen.1009049 33196638 PMC7704051

[jcv270023-bib-0038] Santos, I. S. , Matijasevich, A. , Capilheira, M. F. , Anselmi, L. , & Barros, F. C. (2015). Excessive crying at 3 months of age and behavioural problems at 4 years age: A prospective cohort study. Journal of Epidemiology & Community Health, 69(7), 654–659. 10.1136/jech-2014-204568 25700531 PMC4484259

[jcv270023-bib-0039] Sekine, M. , Chen, X. , Hamanishi, S. , Wang, H. , Yamagami, T. , & Kagamimori, S. (2002). The validity of sleeping hours of healthy young children as reported by their parents. Journal of Epidemiology, 12(3), 237–242. 10.2188/jea.12.237 12164326 PMC10499471

[jcv270023-bib-0040] Simard, V. , Bernier, A. , Belanger, M. E. , & Carrier, J. (2013). Infant attachment and toddlers' sleep assessed by maternal reports and actigraphy: Different measurement methods yield different relations. Journal of Pediatric Psychology, 38(5), 473–483. 10.1093/jpepsy/jst001 23428653

[jcv270023-bib-0041] Smaldone, A. , Honig, J. C. , & Byrne, M. W. (2007). Sleepless in America: Inadequate sleep and relationships to health and well‐being of our nation's children. Pediatrics, 119(Suppl 1), S29–S37. 10.1542/peds.2006-2089F 17272582

[jcv270023-bib-0042] Smarius, L. J. , Strieder, T. G. , Loomans, E. M. , Doreleijers, T. A. , Vrijkotte, T. G. , Gemke, R. J. , & van Eijsden, M. (2017). Excessive infant crying doubles the risk of mood and behavioral problems at age 5: Evidence for mediation by maternal characteristics. European Child & Adolescent Psychiatry, 26(3), 293–302. 10.1007/s00787-016-0888-4 27422707 PMC5323467

[jcv270023-bib-0043] Touchette, E. , Dionne, G. , Forget‐Dubois, N. , Petit, D. , Perusse, D. , Falissard, B. , Tremblay, R. E. , Boivin, M. , & Montplaisir, J. Y. (2013). Genetic and environmental influences on daytime and nighttime sleep duration in early childhood. Pediatrics, 131(6), e1874–e1880. 10.1542/peds.2012-2284 23713101

[jcv270023-bib-0044] Vermillet, A. Q. , Tølbøll, K. , Mizan, S. L. , Skewes, J. C. , & Parsons, C. E. (2022). Crying in the first 12 months of life: A systematic review and meta‐analysis of cross‐country parent‐reported data and modeling of the “cry curve”. Child Development, 93(4), 1201–1222. 10.1111/cdev.13760 35438798 PMC9541248

[jcv270023-bib-0045] Viktorsson, C. , Ronald, A. , & Falck‐Ytter, T. (2024). A comparison of sleep and settle behaviors across twins and singletons at 5 months of age. Retrieved from. https://osf.io/preprints/osf/j4vqe

[jcv270023-bib-0046] Watanabe, K. , Jansen, P. R. , Savage, J. E. , Nandakumar, P. , Wang, X. , Agee, M. , Aslibekyan, S. , Auton, A. , Bell, R. K. , Bryc, K. , Clark, S. K. , Elson, S. L. , Fletez‐Brant, K. , Fontanillas, P. , Furlotte, N. A. , Gandhi, P. M. , Heilbron, K. , Hicks, B. , Huber, K. E. , … Posthuma, D. (2022). Genome‐wide meta‐analysis of insomnia prioritizes genes associated with metabolic and psychiatric pathways. Nature Genetics, 54(8), 1125–1132. 10.1038/s41588-022-01124-w 35835914

[jcv270023-bib-0047] Weinraub, M. , Bender, R. H. , Friedman, S. L. , Susman, E. J. , Knoke, B. , Bradley, R. , Houts, R. , & Williams, J. (2012). Patterns of developmental change in infants' nighttime sleep awakenings from 6 through 36 months of age. Developmental Psychology, 48(6), 1511–1528. 10.1037/a0027680 22448981

[jcv270023-bib-0048] Wolke, D. , Bilgin, A. , & Samara, M. (2017). Systematic review and meta‐analysis: Fussing and crying durations and prevalence of colic in infants. Journal of Pediatrics, 185, 55–61. 10.1016/j.jpeds.2017.02.020 28385295

[jcv270023-bib-0049] Wolke, D. , Rizzo, P. , & Woods, S. (2002). Persistent infant crying and hyperactivity problems in middle childhood. Pediatrics, 109(6), 1054–1060. 10.1542/peds.109.6.1054 12042542

