# Supplementary material for: Genetic and environmental influences on sleep quality, ability to settle, and crying duration in 2‐ and 5‐month‐old infants: A longitudinal twin study
Source: JCPP Adv. 2025 Jul 4;6(1):e70023. doi: 10.1002/jcv2.70023 (PMC12973126; doi:10.1002/jcv2.70023)
Supplement: Supplementary file 1 — Supporting Information S1 [file JCV2-6-e70023-s001.docx]

Supporting Information

**Genetic and environmental influences on sleep quality, ability to settle, and crying duration in 2- and 5-month-old infants: A longitudinal twin study**

Charlotte Viktorsson^1*^, Ashraf Yahia^2,3^, Mark J. Taylor^4^, Angelica Ronald^5^, Kristiina Tammimies^2,3^, & Terje Falck-Ytter^1,2^

^1^Development and Neurodiversity Lab, Department of Psychology, Uppsala University; Uppsala, Sweden

^2^Center of Neurodevelopmental Disorders (KIND), Division of Neuropsychiatry, Department of Women’s and Children’s Health, Karolinska Institutet, Stockholm, Sweden

^3^Astrid Lindgren Children’s Hospital, Karolinska University Hospital, Region Stockholm, Sweden

^4^Department of Medical Epidemiology & Biostatistics, Karolinska Institutet; Stockholm, Sweden

^5^School of Psychology, Faculty of Health and Medical Sciences, University of Surrey; Guildford, UK

*Corresponding author. Email: charlotte.viktorsson@psyk.uu.se

**Supporting Information S1.**

Sample collection, genotyping, and imputation

Saliva samples were collected from the twins at home. The DNA extraction was done at the KI-biobank using the Hamilton ChemagicSTAR® platform. After isolation, DNA was dissolved in 10 mM Tris-HCl buffer, pH 8.0, and concentration was measured.

DNA samples were genotyped for 730,059 single-nucleotide polymorphisms (SNPs) using an Illumina Infinium™ Global Screening Array v3.0 array at the SNP&SEQ Technology Platform at Uppsala University, Uppsala, Sweden. The genotype calls were made using GenomeStudio 2.0.3. Of all the SNPs, 97.41% had a sample call rate > 98%, and the average per-sample call rate was 99.22% (59.92%- 99.62%).

Quality control (QC) of the genotyping data was performed using PLINK 1.9 (Chang et al., 2015). The following QC metrics were used for excluding individuals or SNPS from the analysis: 1) Variants or individuals with genotype missingness > 2%, 2) Variants not in Hardy-Weinberg equilibrium (p < 1 × 10−6), 3) Individuals with heterozygosity rate > ±3 standard deviations from the samples’ heterozygosity rate mean. Furthermore, we checked for sex mismatches using the “--check-sex” PLINK command (X chromosome homozygosity estimate for males > 0.8 and females < 0.2). A total of 866 individuals and 282,921 variants passed our quality filters with a total genotyping rate of 99.9%. Nine individuals (not including a twin pair) were filtered due to high variant missingness, and 28 individuals (including 11 twin pairs) were filtered due to heterozygosity issues.

Population stratification was assessed using the multidimensional scaling (MDS) approach anchored to phase 3 of the 1000 genome project as described by Marees et al., 2018 (Marees et al., 2018). Strand issues, problematic SNPs, and reference correspondence were checked before anchoring. MDS component scores were produced using the “--cluster --mds-plot” PLINK command. We visualized the first two principal components (PCs) to check for outliers. Minor allele frequency (MAF) was set at 1%. The first 10 PCs were used in the statistical analyses to control for the genetic ancestry inference.

Phasing and imputation were performed based on phase 3 of the 1000 genome project using the default parameters of SHAPEIT4 (Delaneau et al., 2019) and IMPUTE5 (Rubinacci et al., 2020), respectively. After imputation and QC an additional 20 individuals (including 7 twin pairs) were filtered due to heterozygosity ±3 standard deviations from the samples’ heterozygosity rate mean. A total of 846 individuals and 8,958,911 variants remained for downstream analysis.

Base data and polygenic risk score calculations

*Selecting base data*

We selected seven genome-wide association studies (GWAS) to serve as base data to calculate polygenic risk scores (PRS) for sleep-related traits and psychiatric conditions. A GWAS on height was added as a control trait GWAS (Yengo et al., 2022). We only used summary statistics of European-ancestry individuals as base data.

| Description of the base GWAS studies used for PRS calculation. *The number of participants indicates the number of European-ancestry individuals we used for PRS calculation **Included summary statistics with and without samples from 23andMe. We only used the summary statistics without 23andMe data. | | | |
| --- | --- | --- | --- |
| Phenotype | GWAS study | Number of participants* | Remarks |
| ADHD | (Demontis et al., 2023) | 51568 |  |
| ASD | (Grove et al., 2019) | 46350 |  |
| Depression | (Howard et al., 2019)** | 500,199 |  |
| Sleep duration | (Austin-Zimmerman et al., 2023) | 445,966 | The report contained two summary statistics, one for long sleep and the other for short sleep; we used both. Multiple ancestries were included, but we used only summary statistics from European ancestry. |
| Circadian rhythm | (Jones et al., 2019)** | 403195 | The report contained two summary statistics, one for a binary phenotype (morning person vs. evening person) and one for a non-binary phenotype. We used the binary phenotype summary statistics. |
| Insomnia | (Watanabe et al., 2022)** | 386,988 |  |
| Anxiety | (Otowa et al., 2016) | 17,310 | Contained two summary statistics, one for the binary phenotype (anxiety vs. no anxiety) and one for a quantitative factor score indexing anxiety disorder liability. We used the binary phenotype summary statistics. |

Base data pre-processing and calculating PRS

We filtered SNPs with MAF < 1% and information imputation score < 0.8 when those parameters were provided. The summary statistics of the sleep duration were provided in chromosome:position:allele1:allele2 format. We used a Python script to convert it into rsIDs. We checked for duplicated and ambiguous SNPs and arranged the base data files per the requirements of PRS-CS (Ge et al., 2019). PRS was calculated using the default parameters of PRS-CS (Ge et al., 2019). We calculated the per individual PRS using the “--score” PLINK command.

Correlation matrix of all polygenic scores

|  | **1** | **2** | **3** | **4** | **5** | **6** | **7** | **8** |
| --- | --- | --- | --- | --- | --- | --- | --- | --- |
| **1 ADHD** | 1 |  |  |  |  |  |  |  |
| **2 Autism** | .289** | 1 |  |  |  |  |  |  |
| **3 Anxiety** | .098** | .047 | 1 |  |  |  |  |  |
| **4 Depression** | .326** | .111** | .115** | 1 |  |  |  |  |
| **5 Circadian rhythm** | -.104** | -.088* | -.033 | -.075* | 1 |  |  |  |
| **6 Insomnia** | .175** | .024 | .035 | .255** | -.070* | 1 |  |  |
| **7 Short sleep** | .258** | .018 | .072* | .262** | -.018 | .383** | 1 |  |
| **8 Long sleep** | .112** | -.022 | -.048 | .143** | -.029 | .065 | .055 | 1 |

* = p <.05, ** = p <.01

Distributional plots of all polygenic scores


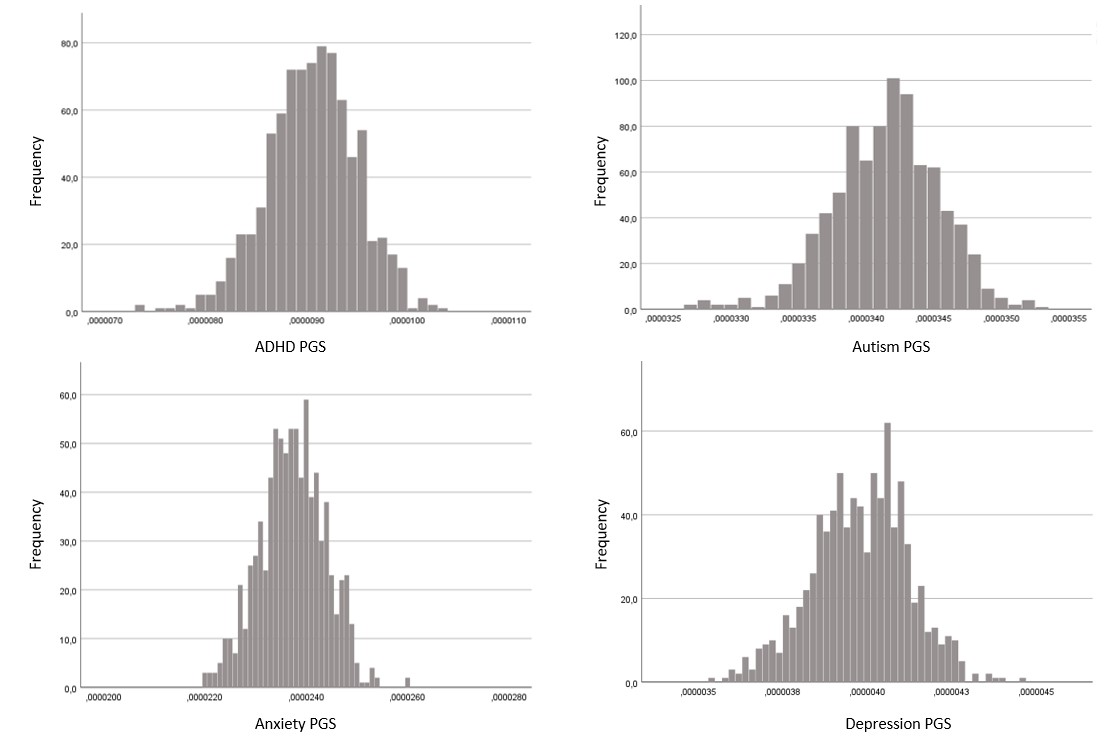


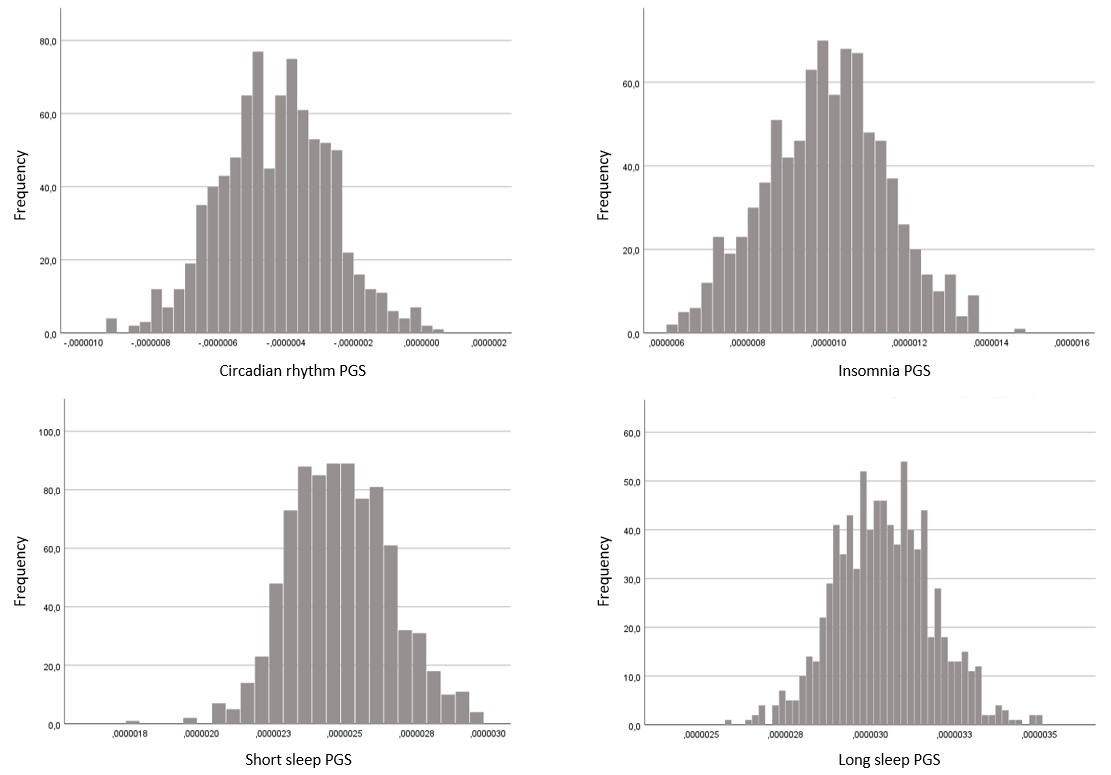


**Supporting Information S2.**

Distributional plots of all variables at 2 and 5 months.


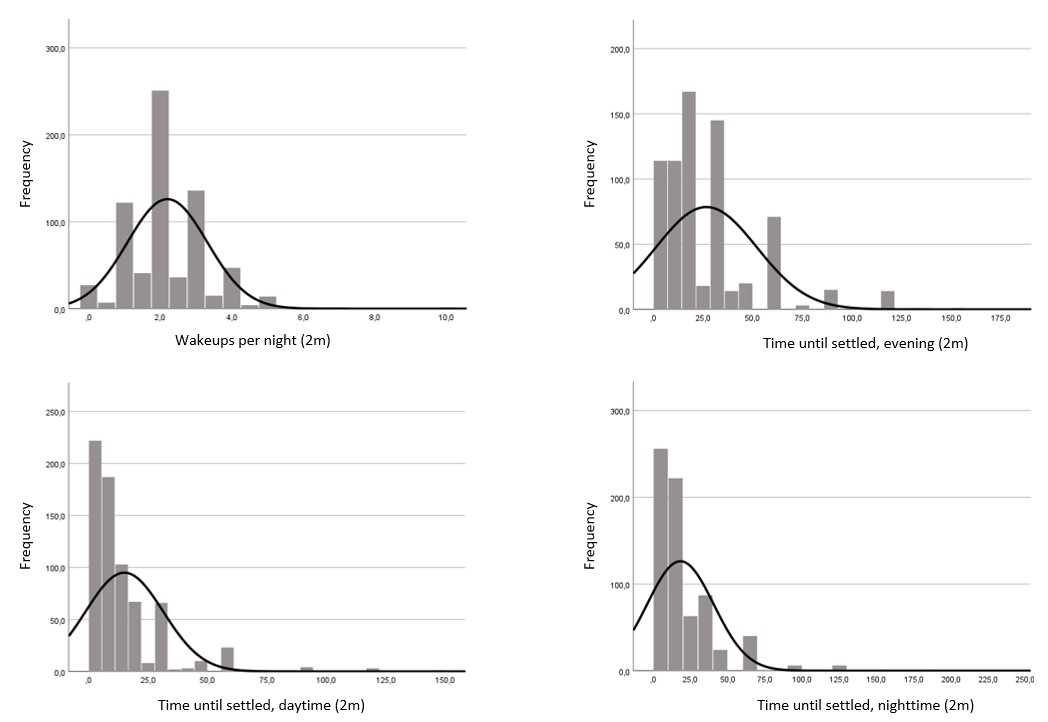


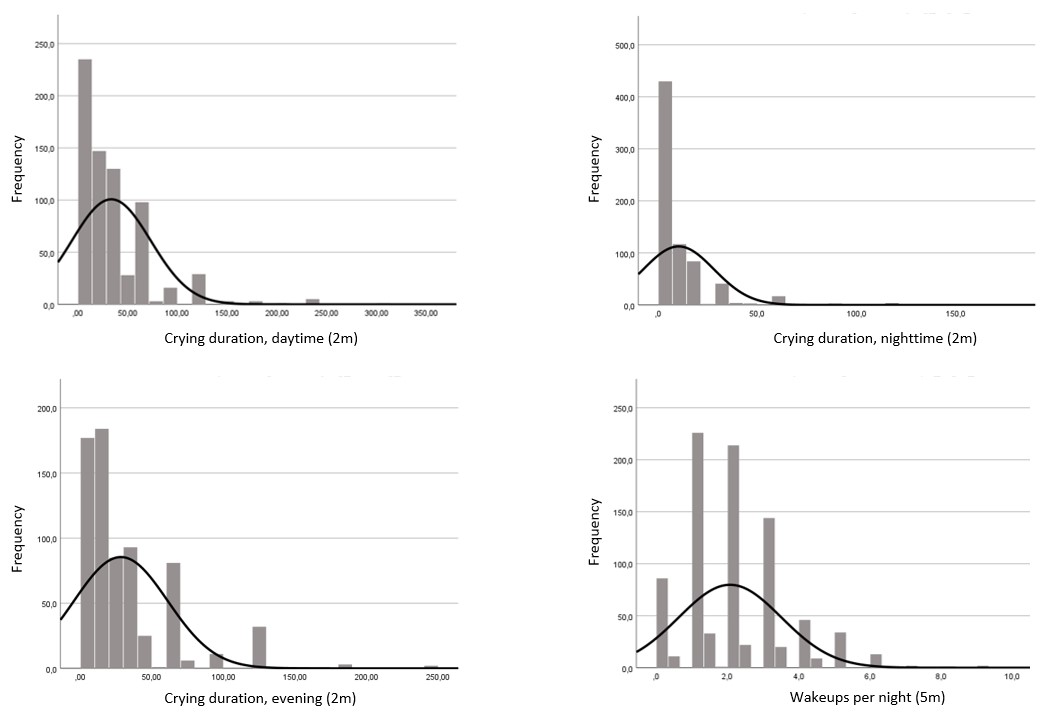


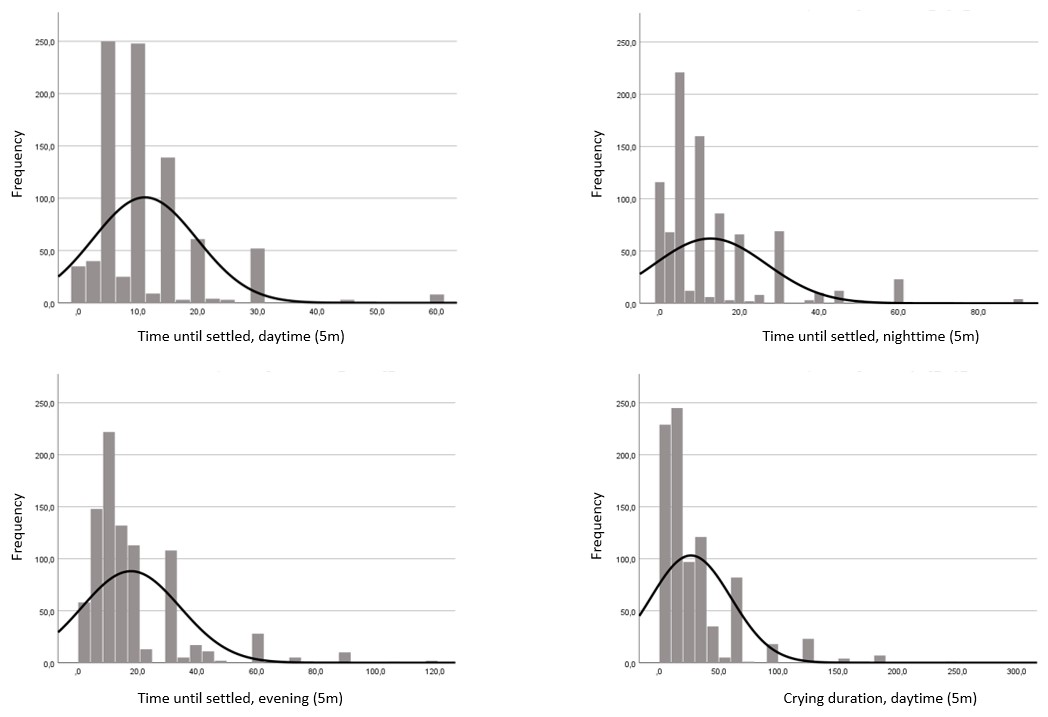


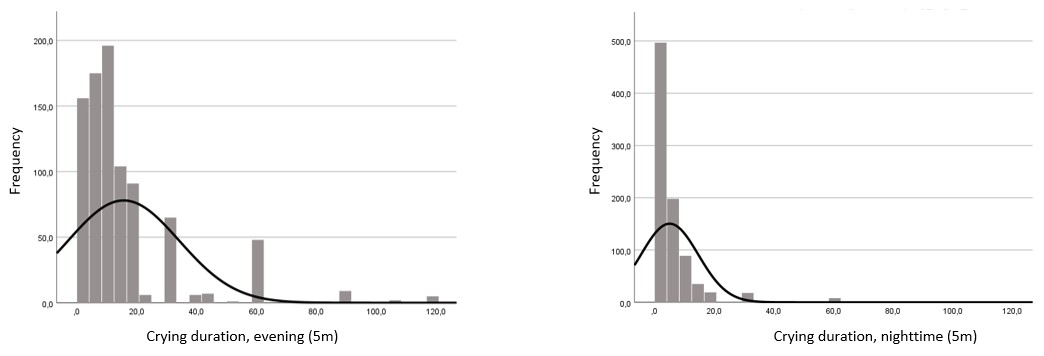


**Supporting Information S3**. Running twin analyses without regressing out covariates.

As requested by a reviewer, we also run all univariate twin analyses without regressing out covariates (other than age and sex). When doing this, the same pattern of met (and unmet) assumptions was found. Likewise, the same models were chosen and the estimates from these models led to the same conclusions as our original findings. See chosen models and estimates in the table below.

|  | **Model** | **-2LL** | **AIC** | **Comparison model** | **Δ χ2** | ***p*** | **A** | **C** | **E** | **s** |
| --- | --- | --- | --- | --- | --- | --- | --- | --- | --- | --- |
| **2 months** |  |  |  |  |  |  | **[95% CI]** | | |  |
| Wakeups per night | ACE | 1580.21 | 1588.21 | ACE-s | 0.01 | 0.91 | .30 [.20; .43] | .61 [.48; .71] | .09 [.07; .12] | - |
| Settle (daytime) | ACE | 1734.68 | 1742.68 | ACE-s | 0.04 | 0.84 | .19 [.02; .38] | .57 [.39; .71] | .24 [.19; .31] | - |
| Settle (evening) | ACE | 1830.68 | 1838.68 | ACE-s | 0.07 | 0.79 | .30 [.06; .55] | .36 [.13; .56] | .34 [.27; .43] | - |
| Settle (nighttime) | CE | 1804.00 | 1810.00 | ACE-s | 1.46 | 0.48 | - | .66 [.60; .71] | .34 [.29; .40] | - |
| Crying (daytime) | AE-s | 1773.94 | 1781.94 | ACE-s | <.001 | 1.00 | .49 [.19; .66] | - | .51 [.34; .81] | .17 [.09; .25] |
| Crying (evening) | ACE | 1755.68 | 1763.68 | ACE-s | 0.15 | 0.69 | .44 [.26; .66] | .35 [.14; .52] | .21 [.17; .27] | - |
| Crying (nighttime) | AE-s | 1891.90 | 1899.90 | ACE-s | <.001 | 1.00 | .45 [.19; .63] | - | .55 [.37; .81] | .16 [.08; .23] |
| **5 months** |  |  |  |  |  |  |  |  |  |  |
| Wakeups per night | ACE | 2143.71 | 2151.71 | ACE-s | 3.52 | 0.06 | .54 [.38; .73] | .28 [.10; .44] | .17 [.14; .21] | - |
| Settle (daytime) | AE | 2345.07 | 2351.07 | ACE-s | 0.003 | 0.99 | .67 [.59; .73] | - | .33 [.27; .41] | - |
| Settle (evening) | AE-s | 2296.24 | 2304.24 | ACE-s | <.001 | 1.00 | .52 [.30; .67] | - | .48 [.33; .70] | .10 [.01; .17] |
| Settle (nighttime) | AE-s | 2309.43 | 2317.43 | ACE-s | <.001 | 1.00 | .51 [.28; .66] | - | .49 [.34; .72] | .09 [.001; .17] |
| Crying (daytime) | AE-s | 2243.61 | 2251.61 | ACE-s | <.001 | 1.00 | .21 [<.01; .43] | - | .79 [.57; 1.00] | .22 [.15; .28] |
| Crying (evening) | AE-s | 2169.99 | 2177.99 | ACE-s | <.001 | 1.00 | .69 [.56; .78] | - | .31 [.22; .44] | .11 [.04; .17] |
| Crying (nighttime) | AE | 2325.29 | 2331.29 | ACE-s | 0.13 | 0.93 | .64 [.56; .70] | - | .36 [.30; .44] | - |

Summary of model fitting for the selected univariate twin models (with only age and sex covariates regressed out).

*-2LL:* *minus 2 log likelihood fit statistic. AIC: Akaike Information Criterion, lower value denotes better model fit.*

*Δ χ2: Change in −2LL statistic between two models, distributed as χ2*

**Tables and Figures**

**Table S1**. GEE analyses of the associations between background variables and sleep/settle behaviors (shaded squares represent statistically significant associations, alpha level .05).

|  | **Standardized β (p-value)** | | | | | | | | | | | | | |
| --- | --- | --- | --- | --- | --- | --- | --- | --- | --- | --- | --- | --- | --- | --- |
|  | **2 months** | | | | | | | **5 months** | | | | | | |
|  | Nighttime awakenings | Time until settled (daytime) | Time until settled (evening) | Time until settled (nighttime) | Crying duration (daytime) | Crying duration (evening) | Crying duration (nighttime) | Nighttime awakenings | Time until settled (daytime) | Time until settled (evening) | Time until settled (nighttime) | Crying duration (daytime) | Crying duration (evening) | Crying duration (nighttime) |
| **Gestational age** | -.136 (.005) | -.020 (.663) | -.070 (.148) | -.055 (.301) | -.119 (.016) | -3.180 (.047) | -.072 (.098) | -.016 (.703) | -.032 (.479) | -.017 (.673) | .032 (.371) | -.078 (.057) | -.067 (.106) | -.049 (.229) |
| **Birthweight** | .018 (.663) | -.009 (.806) | -.061 (.178) | -.058 (.237) | -.073 (.200) | -.106 (.040) | -.040 (.255) | .013 (.745) | -.007 (.858) | -.069 (.101) | .019 (.573) | -.063 (.097) | -.120 (.002) | -.055 (.046) |
| **Maternal age** | .041 (.436) | .035 (.511) | .074 (.151) | -.084 (.100) | -.093 (.027) | -.054 (.288) | -.005 (.923) | -.040 (.365) | .044 (.305) | .031 (.462) | .040 (.366) | -.043 (.264) | .020 (.582) | .009 (.806) |
| **Paternal age** | .039 (.424) | .062 (.208) | .051 (.329) | -.039 (.372) | -.050 (.185) | .002 (.970) | .038 (.445) | .030 (.497) | .035 (.373) | .043 (.295) | .020 (.637) | .005 (.892) | .067 (.109) | .076 (.082) |
| **Daylight exposure** | -.027 (.619) | .037 (.405) | .049 (.302) | -.192 (<.001) | .071 (.086) | .057 (.215) | -.022 (.650) | -.157 (<.001) | -.031 (.455) | -.005 (.910) | -.029 (.494) | -.066 (.142) | -.045 (.281) | -.043 (.176) |
| **Family income** | -.059 (.230) | -.084 (.044) | -.095 (.041) | -.106 (.051) | -.079 (.140) | -.092 (.052) | -.107 (.012) | .009 (.832) | -.067 (.118) | -.035 (.367) | -.033 (.484) | -.143 (.005) | -.065 (.137) | -.007 (.880) |

**Table S2**. Testing twin modeling assumptions

| **2-month assessment** | | |  |  |  | **Comparative fit with saturated model** | | |
| --- | --- | --- | --- | --- | --- | --- | --- | --- |
|  | **Model** | **-2LL** | **#**  **parameters** | ***df*** | **AIC** | **Δ χ2** | **Δ df** | **p** |
| Wakeups per night |  |  |  |  |  |  |  |  |
|  | Fully sat. | 1544.88 | 10 | 695 | 1564.88 | - | - | - |
|  | Submodel 1 | 1547.55 | 8 | 697 | 1563.55 | 2.67 | 2 | 0.26 |
|  | Submodel 2 | 1553.13 | 6 | 699 | 1565.13 | 8.25 | 4 | 0.08 |
|  | Submodel 3 | 1553.27 | 5 | 700 | 1563.27 | 8.40 | 5 | 0.14 |
|  | Submodel 4 | 1553.30 | 4 | 701 | 1561.30 | 8.42 | 6 | 0.21 |
| Settle day |  |  |  |  |  |  |  |  |
|  | Fully sat. | 1704.27 | 10 | 684 | 1724.27 | - | - | - |
|  | Submodel 1 | 1707.56 | 8 | 686 | 1723.56 | 3.29 | 2 | 0.19 |
|  | Submodel 2 | 1739.16 | 6 | 688 | 1751.16 | 34.89 | 4 | <.001 |
|  | Submodel 3 | 1739.34 | 5 | 689 | 1749.34 | 35.07 | 5 | <.001 |
|  | Submodel 4 | 1739.37 | 4 | 690 | 1747.37 | 35.10 | 6 | <.001 |
| Settle evening |  |  |  |  |  |  |  |  |
|  | Fully sat. | 1724.31 | 10 | 684 | 1744.31 | - | - | - |
|  | Submodel 1 | 1728.78 | 8 | 686 | 1744.78 | 4.47 | 2 | 0.11 |
|  | Submodel 2 | 1740.72 | 6 | 688 | 1752.72 | 16.41 | 4 | 0.003 |
|  | Submodel 3 | 1740.77 | 5 | 689 | 1750.77 | 16.46 | 5 | 0.006 |
|  | Submodel 4 | 1740.89 | 4 | 690 | 1748.89 | 16.58 | 6 | 0.01 |
| Settle night |  |  |  |  |  |  |  |  |
|  | Fully sat. | 1761.58 | 10 | 698 | 1781.58 | - | - | - |
|  | Submodel 1 | 1762.86 | 8 | 700 | 1778.86 | 1.28 | 2 | 0.53 |
|  | Submodel 2 | 1772.63 | 6 | 702 | 1784.63 | 11.05 | 4 | 0.03 |
|  | Submodel 3 | 1772.75 | 5 | 703 | 1782.75 | 11.17 | 5 | 0.05 |
|  | Submodel 4 | 1789.60 | 4 | 704 | 1797.60 | 28.02 | 6 | <.001 |
| Crying day |  |  |  |  |  |  |  |  |
|  | Fully sat. | 1758.29 | 10 | 688 | 1778.29 | - | - | - |
|  | Submodel 1 | 1767.06 | 8 | 690 | 1783.06 | 8.77 | 2 | 0.01 |
|  | Submodel 2 | 1785.10 | 6 | 692 | 1797.10 | 26.81 | 4 | <.001 |
|  | Submodel 3 | 1785.30 | 5 | 693 | 1795.30 | 27.01 | 5 | <.001 |
|  | Submodel 4 | 1786.32 | 4 | 694 | 1794.32 | 28.03 | 6 | <.001 |
| Crying evening |  |  |  |  |  |  |  |  |
|  | Fully sat. | 1687.68 | 10 | 677 | 1707.68 | - | - | - |
|  | Submodel 1 | 1689.49 | 8 | 679 | 1705.49 | 1.82 | 2 | 0.40 |
|  | Submodel 2 | 1690.42 | 6 | 681 | 1702.42 | 2.74 | 4 | 0.60 |
|  | Submodel 3 | 1692.03 | 5 | 682 | 1702.03 | 4.35 | 5 | 0.50 |
|  | Submodel 4 | 1692.04 | 4 | 683 | 1700.04 | 4.37 | 6 | 0.63 |
| Crying night |  |  |  |  |  |  |  |  |
|  | Fully sat. | 1799.51 | 10 | 692 | 1819.51 | - | - | - |
|  | Submodel 1 | 1805.55 | 8 | 694 | 1821.55 | 6.03 | 2 | 0.05 |
|  | Submodel 2 | 1854.18 | 6 | 696 | 1866.18 | 54.67 | 4 | <.001 |
|  | Submodel 3 | 1855.55 | 5 | 697 | 1865.55 | 56.04 | 5 | <.001 |
|  | Submodel 4 | 1879.44 | 4 | 698 | 1887.44 | 79.93 | 6 | <.001 |
| **5-month assessment** | |  |  |  |  | **Comparative fit with saturated model** | | |
|  | **Model** | **-2LL** | **#**  **parameters** | ***df*** | **AIC** | **Δ χ2** | **Δ df** | **p** |
| Wakeups per night |  |  |  |  |  |  |  |  |
|  | Fully sat. | 2157.19 | 10 | 855 | 2177.19 | - | - | - |
|  | Submodel 1 | 2161.36 | 8 | 857 | 2177.36 | 4.17 | 2 | 0.12 |
|  | Submodel 2 | 2165.58 | 6 | 859 | 2177.58 | 8.39 | 4 | 0.08 |
|  | Submodel 3 | 2165.60 | 5 | 860 | 2175.60 | 8.41 | 5 | 0.13 |
|  | Submodel 4 | 2170.45 | 4 | 861 | 2178.45 | 13.26 | 6 | 0.04 |
| Settle day |  |  |  |  |  |  |  |  |
|  | Fully sat. | 2184.97 | 10 | 870 | 2204.97 | - | - | - |
|  | Submodel 1 | 2191.77 | 8 | 872 | 2207.77 | 6.81 | 2 | 0.03 |
|  | Submodel 2 | 2201.97 | 6 | 874 | 2213.97 | 17.00 | 4 | 0.002 |
|  | Submodel 3 | 2202.39 | 5 | 875 | 2212.39 | 17.42 | 5 | 0.004 |
|  | Submodel 4 | 2202.73 | 4 | 876 | 2210.73 | 17.76 | 6 | 0.007 |
| Settle evening |  |  |  |  |  |  |  |  |
|  | Fully sat. | 2261.93 | 10 | 865 | 2281.93 | - | - | - |
|  | Submodel 1 | 2266.42 | 8 | 867 | 2282.42 | 4.49 | 2 | 0.11 |
|  | Submodel 2 | 2277.00 | 6 | 869 | 2289.00 | 15.07 | 4 | 0.005 |
|  | Submodel 3 | 2277.66 | 5 | 870 | 2287.66 | 15.73 | 5 | 0.008 |
|  | Submodel 4 | 2282.37 | 4 | 871 | 2290.37 | 20.44 | 6 | 0.002 |
| Settle night |  |  |  |  |  |  |  |  |
|  | Fully sat. | 2262.82 | 10 | 864 | 2282.82 | - | - | - |
|  | Submodel 1 | 2264.91 | 8 | 866 | 2280.91 | 2.09 | 2 | 0.35 |
|  | Submodel 2 | 2274.44 | 6 | 868 | 2286.44 | 11.62 | 4 | 0.02 |
|  | Submodel 3 | 2274.66 | 5 | 869 | 2284.66 | 11.84 | 5 | 0.04 |
|  | Submodel 4 | 2281.41 | 4 | 870 | 2289.41 | 18.59 | 6 | 0.005 |
| Crying day |  |  |  |  |  |  |  |  |
|  | Fully sat. | 2171.78 | 10 | 848 | 2191.78 | - | - | - |
|  | Submodel 1 | 2174.98 | 8 | 850 | 2190.98 | 3.21 | 2 | 0.20 |
|  | Submodel 2 | 2200.94 | 6 | 852 | 2212.94 | 29.16 | 4 | <.001 |
|  | Submodel 3 | 2201.49 | 5 | 853 | 2211.49 | 29.71 | 5 | <.001 |
|  | Submodel 4 | 2215.73 | 4 | 854 | 2223.73 | 43.95 | 6 | <.001 |
| Crying evening |  |  |  |  |  |  |  |  |
|  | Fully sat. | 2110.72 | 10 | 840 | 2130.72 | - | - | - |
|  | Submodel 1 | 2117.61 | 8 | 842 | 2133.61 | 6.88 | 2 | 0.03 |
|  | Submodel 2 | 2144.30 | 6 | 844 | 2156.30 | 33.58 | 4 | <.001 |
|  | Submodel 3 | 2144.67 | 5 | 845 | 2154.67 | 33.95 | 5 | <.001 |
|  | Submodel 4 | 2151.46 | 4 | 846 | 2159.46 | 40.74 | 6 | <.001 |
| Crying night |  |  |  |  |  |  |  |  |
|  | Fully sat. | 2001.65 | 10 | 838 | 2021.65 | - | - | - |
|  | Submodel 1 | 2011.53 | 8 | 840 | 2027.53 | 9.88 | 2 | 0.007 |
|  | Submodel 2 | 2080.13 | 6 | 842 | 2092.13 | 78.48 | 4 | <.001 |
|  | Submodel 3 | 2080.97 | 5 | 843 | 2090.97 | 79.32 | 5 | <.001 |
|  | Submodel 4 | 2087.95 | 4 | 844 | 2095.95 | 86.30 | 6 | <.001 |

The fully saturated model is the baseline model, which models the means and variances separately for each twin in a pair and across zygosity.

Submodel 1: Equating means across twins within a pair

Submodel 2:  Equating means across zygosity

Submodel 3: Equating variances across twins within a pair

Submodel 4: Equating variances across zygosity

-2LL: Fit statistic, the lower the better fitting is the model

*df*: Degrees of freedom

AIC: An alternative fit index, lower value denotes better model fit

Δ χ2: Difference in −2LL statistic between two models, distributed χ2

Δ *df:*Difference in degrees of freedom between two models

**Table S3**. Univariate twin model fitting for the number of wakeups per night (at 2 months).

| **Wakeups per night (2 months)** | | |  |  |  |  |  |  |  |  |  |  |
| --- | --- | --- | --- | --- | --- | --- | --- | --- | --- | --- | --- | --- |
| **Model** | **-2LL** | **# parameters** | ***df*** | **AIC** | **Comparison model** | **Δ χ2** | **Δ *df*** | ***p*** | **A** | **C (95% CI)** | **E** | **s** |
| Fully sat. | 1544.88 | 10 | 695 | 1564.88 | **-** | **-** | **-** | **-** | **-** | **-** | **-** | **-** |
| ACE-s | 1553.27 | 5 | 700 | 1563.27 | Fully sat. | 8.40 | 5 | .14 | .37 [.02; .83] | .51 [<.001; .97] | .11 [.008; .33] | .06 [-.99; .25] |
| ACE | 1553.30 | 4 | 701 | 1561.30 | ACE-s | 0.02 | 1 | .88 | .30 [.20; .43] | .61 [.48; .71] | .09 [.07; .12] | - |
| AE-s | 1554.15 | 4 | 701 | 1562.15 | ACE-s | 0.87 | 1 | .35 | .77 [.66; .84] | - | .21 [.16; .26] | .21 [.16; .26] |
| AE | 1596.45 | 3 | 702 | 1602.45 | ACE-s | 43.18 | 2 | <.001 | .91 [.88; .93] | - | .09 [.07; .12] | - |
| CE | 1593.49 | 3 | 702 | 1599.49 | ACE-s | 40.21 | 2 | <.001 | - | .84 [.80; .87] | .16 [.13; .20] | - |
| E | 1974.33 | 2 | 703 | 1978.33 | ACE-s | 421.05 | 3 | <.001 | - | - | 1 | - |

**Table S4**. Univariate twin model fitting

| **Settle day (2 months)** | | |  |  |  |  |  |  |  |  |  |  |
| --- | --- | --- | --- | --- | --- | --- | --- | --- | --- | --- | --- | --- |
| **Model** | **-2LL** | **# parameters** | ***df*** | **AIC** | **Comparison model** | **Δ χ2** | **Δ *df*** | ***p*** | **A** | **C (95% CI)** | **E** | **s** |
| Fully sat. | 1704.27 | 10 | 684 | 1724.27 | **-** | **-** | **-** | **-** | **-** | **-** | **-** | **-** |
| ACE-s | 1739.34 | 5 | 689 | 1749.34 | Fully sat. | 35.07 | 5 | <.001 | .12 [.002; .61] | .73 [<.001; .96] | .15 [.02; .95] | -.16 [-.99; .29] |
| ACE | 1739.37 | 4 | 690 | 1747.37 | ACE-s | 0.02 | 1 | 0.88 | .19 [.02; .38] | .56 [.39; .71] | .25 [.20; .31] | - |
| AE-s | 1740.06 | 4 | 690 | 1748.06 | ACE-s | 0.72 | 1 | 0.40 | .41 [.004; .62] | - | .59 [.38; .99] | .22 [.14; .30] |
| AE | 1765.95 | 3 | 691 | 1771.95 | ACE-s | 26.61 | 2 | <.001 | .76 [.71; .81] | - | .23 [.19; .29] | - |
| CE | 1744.00 | 3 | 691 | 1750.00 | ACE-s | 4.66 | 2 | 0.10 | - | .71 [.65; .76] | .29 [.24; .35] | - |
| E | 1982.68 | 2 | 692 | 1986.68 | ACE-s | 243.33 | 3 | <.001 | - | - | 1 | - |

**Table S5**. Univariate twin model fitting

| **Settle evening (2 months)** | | |  |  |  |  |  |  |  |  |  |  |
| --- | --- | --- | --- | --- | --- | --- | --- | --- | --- | --- | --- | --- |
| **Model** | **-2LL** | **# parameters** | ***df*** | **AIC** | **Comparison model** | **Δ χ2** | **Δ *df*** | ***p*** | **A** | **C (95% CI)** | **E** | **s** |
| Fully sat. | 1724.31 | 10 | 684 | 1744.31 | **-** | **-** | **-** | **-** | **-** | **-** | **-** | **-** |
| ACE-s | 1740.77 | 5 | 689 | 1750.77 | Fully sat. | 16.46 | 5 | 0.01 | .15 [.008; .63] | .69 [<.001; .94] | .16 [.04; .88] | -.27 [-.99; .22] |
| ACE | 1740.89 | 4 | 690 | 1748.89 | ACE-s | 0.13 | 1 | 0.72 | .29 [.05; .55] | .36 [.13; .56] | .34 [.27; .43] | - |
| AE-s | 1741.51 | 4 | 690 | 1749.51 | ACE-s | 0.74 | 1 | 0.39 | .45 [.07; .64] | - | .55 [.36; .93] | .14 [.04; .23] |
| AE | 1749.33 | 3 | 691 | 1755.33 | ACE-s | 8.56 | 2 | 0.01 | .67 [.60; .74] | - | .33 [.26; .40] | - |
| CE | 1746.61 | 3 | 691 | 1752.61 | ACE-s | 5.84 | 2 | 0.05 | - | .58 [.51; .65] | .42 [.35; .49] | - |
| E | 1888.43 | 2 | 692 | 1892.43 | ACE-s | 147.66 | 3 | <.001 | - | - | 1 | - |

**Table S6**. Univariate twin model fitting

| **Settle night (2 months)** | | |  |  |  |  |  |  |  |  |  |  |
| --- | --- | --- | --- | --- | --- | --- | --- | --- | --- | --- | --- | --- |
| **Model** | **-2LL** | **# parameters** | ***df*** | **AIC** | **Comparison model** | **Δ χ2** | **Δ *df*** | ***p*** | **A** | **C (95% CI)** | **E** | **s** |
| Fully sat. | 1761.58 | 10 | 698 | 1781.58 | **-** | **-** | **-** | **-** | **-** | **-** | **-** | **-** |
| ACE-s | 1788.28 | 5 | 703 | 1798.28 | Fully sat. | 26.70 | 5 | <.001 | .18 [<.001; .42] | <.001 [<.001; .96] | .82 [.04; 1.00] | .23 [-.99; .29] |
| ACE | 1789.82 | 4 | 704 | 1797.82 | ACE-s | 1.54 | 1 | 0.21 | <.001 [<.001; .17] | .65 [.49; .70] | .35 [.29; .42] | - |
| AE-s | 1788.28 | 4 | 704 | 1796.28 | ACE-s | <.001 | 1 | 1.00 | .18 [<.001; .42] | - | .82 [.58; 1.00] | .23 [.16; .29] |
| AE | 1818.12 | 3 | 705 | 1824.12 | ACE-s | 29.83 | 2 | 0.33 | .66 [.59; .71] | - | .34 [.29; .41] | - |
| CE | 1789.82 | 3 | 705 | 1795.82 | ACE-s | 1.54 | 2 | 0.46 | - | .65 [.58; .70] | .35 [.30; .42] | - |
| E | 1982.49 | 2 | 706 | 1986.49 | ACE-s | 194.21 | 3 | <.001 | - | - | 1 | - |

**Table S7**. Univariate twin model fitting

| **Crying day (2 months)** | | |  |  |  |  |  |  |  |  |  |  |
| --- | --- | --- | --- | --- | --- | --- | --- | --- | --- | --- | --- | --- |
| **Model** | **-2LL** | **# parameters** | ***df*** | **AIC** | **Comparison model** | **Δ χ2** | **Δ *df*** | ***p*** | **A** | **C (95% CI)** | **E** | **s** |
| Fully sat. | 1758.29 | 10 | 688 | 1778.29 | **-** | **-** | **-** | **-** | **-** | **-** | **-** | **-** |
| ACE-s | 1785.42 | 5 | 693 | 1795.42 | Fully sat. | 27.13 | 5 | <.001 | .48 [.01; .66] | <.001 [<.001; .95] | .52 [.03; .82] | .17 [-.99; .25] |
| ACE | 1786.32 | 4 | 694 | 1794.32 | ACE-s | 0.90 | 1 | 0.34 | .27 [.07; .49] | .46 [.25; .63] | .27 [.21; .34] | - |
| AE-s | 1785.42 | 4 | 694 | 1793.42 | ACE-s | <.001 | 1 | 1.00 | .48 [.18; .66] | - | .52 [.34; .82] | .17 [.09; .25] |
| AE | 1801.27 | 3 | 695 | 1807.27 | ACE-s | 15.85 | 2 | <.001 | .74 [.68; .79] | - | .26 [.21; .32] | - |
| CE | 1793.36 | 3 | 695 | 1799.36 | ACE-s | 7.94 | 2 | 0.02 | - | .67 [.60; .72] | .33 [.28; .40] | - |
| E | 1990.40 | 2 | 696 | 1994.40 | ACE-s | 204.98 | 3 | <.001 | - | - | 1 | - |

**Table S8**. Univariate twin model fitting

| **Crying evening (2 months)** | | |  |  |  |  |  |  |  |  |  |  |
| --- | --- | --- | --- | --- | --- | --- | --- | --- | --- | --- | --- | --- |
| **Model** | **-2LL** | **# parameters** | ***df*** | **AIC** | **Comparison model** | **Δ χ2** | **Δ *df*** | ***p*** | **A** | **C (95% CI)** | **E** | **s** |
| Fully sat. | 1687.68 | 10 | 677 | 1707.68 | **-** | **-** | **-** | **-** | **-** | **-** | **-** | **-** |
| ACE-s | 1692.03 | 5 | 682 | 1702.03 | Fully sat. | 4.35 | 5 | 0.50 | .48 [.03; .77] | .27 [<.001; .94] | .25 [.02; .52] | .03 [-.99; .99] |
| ACE | 1692.04 | 4 | 683 | 1700.04 | ACE-s | 0.01 | 1 | 0.91 | .44 [.24; .67] | .34 [.12; .52] | .22 [.17; .28] | - |
| AE-s | 1692.27 | 4 | 683 | 1700.27 | ACE-s | 0.24 | 1 | 0.63 | .66 [.47; .77] | - | .34 [.23; .53] | .12 [.04; .19] |
| AE | 1700.44 | 3 | 684 | 1706.44 | ACE-s | 8.41 | 2 | 0.01 | .78 [.73; .83] | - | .22 [.17; .27] | - |
| CE | 1711.64 | 3 | 684 | 1717.64 | ACE-s | 19.61 | 2 | <.001 | - | .67 [.60; .72] | .33 [.28; .40] | - |
| E | 1902.19 | 2 | 685 | 1906.19 | ACE-s | 210.16 | 3 | <.001 | - | - | 1 | - |

**Table S9**. Univariate twin model fitting

| **Crying night (2 months)** | | |  |  |  |  |  |  |  |  |  |  |
| --- | --- | --- | --- | --- | --- | --- | --- | --- | --- | --- | --- | --- |
| **Model** | **-2LL** | **# parameters** | ***df*** | **AIC** | **Comparison model** | **Δ χ2** | **Δ *df*** | ***p*** | **A** | **C (95% CI)** | **E** | **s** |
| Fully sat. | 1799.51 | 10 | 692 | 1819.51 | **-** | **-** | **-** | **-** | **-** | **-** | **-** | **-** |
| ACE-s | 1874.29 | 5 | 697 | 1884.29 | Fully sat. | 74.78 | 5 | <.001 | .46 [20; .64] | <.001 [<.001; .32] | .54 [.35; .80] | .16 [.05; .23] |
| ACE | 1879.44 | 4 | 698 | 1887.44 | ACE-s | 5.15 | 1 | 0.02 | .27 [.03; .55] | .42 [.15; .62] | .31 [.24; .40] | - |
| AE-s | 1874.29 | 4 | 698 | 1882.29 | ACE-s | <.001 | 1 | 1.00 | .46 [.20; .64] | - | .54 [.36; .80] | .16 [.08; .23] |
| AE | 1887.74 | 3 | 699 | 1893.74 | ACE-s | 0.13 | 2 | 0.001 | .70 [.62; .76] | - | .30 [.24; .38] | - |
| CE | 1884.19 | 3 | 699 | 1890.19 | ACE-s | 9.90 | 2 | 0.01 | - | .62 [.54; .69] | .38 [.31; .46] | - |
| E | 1986.47 | 2 | 700 | 1990.47 | ACE-s | 112.18 | 3 | <.001 | - | - | 1 | - |

**Table S10**. Univariate twin model fitting

| **Wakeups per night (5 months)** | | |  |  |  |  |  |  |  |  |  |  |
| --- | --- | --- | --- | --- | --- | --- | --- | --- | --- | --- | --- | --- |
| **Model** | **-2LL** | **# parameters** | ***df*** | **AIC** | **Comparison model** | **Δ χ2** | **Δ *df*** | ***p*** | **A** | **C (95% CI)** | **E** | **s** |
| Fully sat. | 2157.19 | 10 | 855 | 2177.19 | **-** | **-** | **-** | **-** | **-** | **-** | **-** | **-** |
| ACE-s | 2166.73 | 5 | 860 | 2176.73 | Fully sat. | 9.54 | 5 | 0.09 | .08 [.05; .60] | .90 [.22; .92] | .02 [.02; .18] | -.99 [-.99; .007] |
| ACE | 2170.45 | 4 | 861 | 2178.45 | ACE-s | 3.72 | 1 | 0.05 | .56 [.40; .76] | .26 [.07; .42] | .18 [.14; .22] | - |
| AE-s | 2173.04 | 4 | 861 | 2181.04 | ACE-s | 6.31 | 1 | 0.01 | .77 [.68; .84] | - | .23 [.16; .32] | .07 [.005; .14] |
| AE | 2177.40 | 3 | 862 | 2183.40 | ACE-s | 10.68 | 2 | 0.005 | .83 [.79; .86] | - | .17 [.14; .21] | - |
| CE | 2218.30 | 3 | 862 | 2224.30 | ACE-s | 51.57 | 2 | <.001 | - | .68 [.62; .72] | .32 [.28; .38] | - |
| E | 2469.51 | 2 | 863 | 2473.51 | ACE-s | 302.79 | 3 | <.001 | - | - | 1 | - |

**Table S11**. Univariate twin model fitting

| **Settle day (5 months)** | | |  |  |  |  |  |  |  |  |  |  |
| --- | --- | --- | --- | --- | --- | --- | --- | --- | --- | --- | --- | --- |
| **Model** | **-2LL** | **# parameters** | ***df*** | **AIC** | **Comparison model** | **Δ χ2** | **Δ *df*** | ***p*** | **A** | **C (95% CI)** | **E** | **s** |
| Fully sat. | 2184.97 | 10 | 870 | 2204.97 | **-** | **-** | **-** | **-** | **-** | **-** | **-** | **-** |
| ACE-s | 2202.73 | 5 | 875 | 2212.73 | Fully sat. | 17.76 | 5 | 0.007 | .66 [.09; .76] | <.001 [<.001; .86] | .34 [.05; .49] | .004 [-.99; .99] |
| ACE | 2202.74 | 4 | 876 | 2210.74 | ACE-s | 0.008 | 1 | 0.93 | .67 [.42; .73] | <.001 [<.001; .22] | .33 [.27; .41] | - |
| AE-s | 2202.73 | 4 | 876 | 2210.73 | ACE-s | <.001 | 1 | 1.00 | .66 [.51; .76] | - | .34 [.24; .49] | .004 [-.08; .99] |
| AE | 2202.74 | 3 | 877 | 2208.74 | ACE-s | 0.008 | 2 | 0.99 | .67 [.59; .73] | - | .33 [.27; .41] | - |
| CE | 2230.93 | 3 | 877 | 2236.93 | ACE-s | 28.20 | 2 | <.001 | - | .50 [.43; .57] | .50 [.43; .57] | - |
| E | 2351.50 | 2 | 878 | 2355.50 | ACE-s | 148.77 | 3 | <.001 | - | - | 1 | - |

**Table S12**. Univariate twin model fitting

| **Settle evening (5 months)** | | |  |  |  |  |  |  |  |  |  |  |
| --- | --- | --- | --- | --- | --- | --- | --- | --- | --- | --- | --- | --- |
| **Model** | **-2LL** | **# parameters** | ***df*** | **AIC** | **Comparison model** | **Δ χ2** | **Δ *df*** | ***p*** | **A** | **C (95% CI)** | **E** | **s** |
| Fully sat. | 2261.93 | 10 | 865 | 2281.93 | **-** | **-** | **-** | **-** | **-** | **-** | **-** | **-** |
| ACE-s | 2280.64 | 5 | 870 | 2290.64 | Fully sat. | 18.71 | 5 | 0.002 | .52 [22; .67] | <.001 [<.001; .57] | .48 [.21; .70] | .10 [-.19; .17] |
| ACE | 2282.37 | 4 | 871 | 2290.37 | ACE-s | 1.73 | 1 | 0.19 | .43 [.19; .68] | .23 [<.001; .44] | .35 [.29; .42] | - |
| AE-s | 2280.64 | 4 | 871 | 2288.64 | ACE-s | <.001 | 1 | 1.00 | .52 [.30; .67] | - | .48 [.33; .70] | .10 [.01; .17] |
| AE | 2285.57 | 3 | 872 | 2291.57 | ACE-s | 4.93 | 2 | 0.08 | .66 [.60; .72] | - | .34 [.28; .40] | - |
| CE | 2295.51 | 3 | 872 | 2301.51 | ACE-s | 14.87 | 2 | <.001 | - | .57 [.50; .63] | .43 [.37; .50] | - |
| E | 2464.78 | 2 | 873 | 2468.78 | ACE-s | 184.14 | 3 | <.001 | - | - | 1 | - |

**Table S13**. Univariate twin model fitting

| **Settle night (5 months)** | | |  |  |  |  |  |  |  |  |  |  |
| --- | --- | --- | --- | --- | --- | --- | --- | --- | --- | --- | --- | --- |
| **Model** | **-2LL** | **# parameters** | ***df*** | **AIC** | **Comparison model** | **Δ χ2** | **Δ *df*** | ***p*** | **A** | **C (95% CI)** | **E** | **s** |
| Fully sat. | 2262.82 | 10 | 864 | 2281.93 | **-** | **-** | **-** | **-** | **-** | **-** | **-** | **-** |
| ACE-s | 2279.50 | 5 | 869 | 2290.64 | Fully sat. | 16.68 | 5 | 0.005 | .51 [25; .66] | <.001 [<.001; .48] | .49 [.25; .71] | .09 [-.12; .17] |
| ACE | 2281.41 | 4 | 870 | 2290.37 | ACE-s | 1.91 | 1 | 0.005 | .44 [.19; .68] | .20 [<.001; .42] | .36 [.30; .44] | - |
| AE-s | 2279.50 | 4 | 870 | 2288.64 | ACE-s | <.001 | 1 | 1.00 | .51 [.29; .66] | - | .49 [.34; .71] | .09 [.001; .17] |
| AE | 2283.47 | 3 | 871 | 2291.57 | ACE-s | 3.97 | 2 | 0.14 | .64 [.57; .70] | - | .36 [.30; .43] | - |
| CE | 2293.78 | 3 | 871 | 2299.78 | ACE-s | 14.27 | 2 | <.001 | - | .55 [.48; .61] | .45 [.39; .52] | - |
| E | 2447.74 | 2 | 872 | 2468.78 | ACE-s | 168.24 | 3 | <.001 | - | - | 1 | - |

**Table S14**. Univariate twin model fitting

| **Crying day (5 months)** | | |  |  |  |  |  |  |  |  |  |  |
| --- | --- | --- | --- | --- | --- | --- | --- | --- | --- | --- | --- | --- |
| **Model** | **-2LL** | **# parameters** | ***df*** | **AIC** | **Comparison model** | **Δ χ2** | **Δ *df*** | ***p*** | **A** | **C (95% CI)** | **E** | **s** |
| Fully sat. | 2171.78 | 10 | 848 | 2191.78 | **-** | **-** | **-** | **-** | **-** | **-** | **-** | **-** |
| ACE-s | 2212.23 | 5 | 853 | 2222.23 | Fully sat. | 40.45 | 5 | <.001 | .27 [02; .50] | <.001 [<.001; .95] | .71 [.27; .98] | .21 [-.99; .27] |
| ACE | 2215.73 | 4 | 854 | 2223.73 | ACE-s | 3.50 | 1 | 0.06 | .09 [<.001; .30] | .57 [.38; .69] | .34 [.28; .41] | - |
| AE-s | 2212.23 | 4 | 854 | 2220.23 | ACE-s | <.001 | 1 | 1.00 | .29 [.02; .50] | - | .71 [.50; .98] | .21 [.14; .27] |
| AE | 2238.94 | 3 | 855 | 2244.94 | ACE-s | 26.71 | 2 | <.001 | .67 [.61; .73] | - | .33 [.27; .39] | - |
| CE | 2216.53 | 3 | 855 | 2222.53 | ACE-s | 4.30 | 2 | 0.12 | - | .64 [.58; .69] | .36 [.31; .42] | - |
| E | 2444.22 | 2 | 856 | 2448.22 | ACE-s | 232.00 | 3 | <.001 | - | - | 1 | - |

**Table S15**. Univariate twin model fitting

| **Crying evening (5 months)** | | |  |  |  |  |  |  |  |  |  |  |
| --- | --- | --- | --- | --- | --- | --- | --- | --- | --- | --- | --- | --- |
| **Model** | **-2LL** | **# parameters** | ***df*** | **AIC** | **Comparison model** | **Δ χ2** | **Δ *df*** | ***p*** | **A** | **C (95% CI)** | **E** | **s** |
| Fully sat. | 2110.72 | 10 | 840 | 2130.72 | **-** | **-** | **-** | **-** | **-** | **-** | **-** | **-** |
| ACE-s | 2149.05 | 5 | 845 | 2159.05 | Fully sat. | 38.33 | 5 | <.001 | .70 [41; .78] | <.001 [<.001; .40] | .30 [.18; .44] | .09 [-.07; .16] |
| ACE | 2151.46 | 4 | 846 | 2159.46 | ACE-s | 2.42 | 1 | 0.11 | .55 [.35; .79] | .23 [<.001; .41] | .22 [.18; .27] | - |
| AE-s | 2149.05 | 4 | 846 | 2157.05 | ACE-s | <.001 | 1 | 1.00 | .70 [.56; .78] | - | .30 [.22; .44] | .09 [.02; .16] |
| AE | 2155.20 | 3 | 847 | 2161.20 | ACE-s | 6.15 | 2 | 0.05 | .78 [.73; .82] | - | .22 [.18; .27] | - |
| CE | 2185.51 | 3 | 847 | 2191.51 | ACE-s | 36.46 | 2 | <.001 | - | .66 [.60; .71] | .34 [.29; .40] | - |
| E | 2414.05 | 2 | 848 | 2418.05 | ACE-s | 265.00 | 3 | <.001 | - | - | 1 | - |

**Table S16**. Univariate twin model fitting

| **Crying night (5 months)** | | |  |  |  |  |  |  |  |  |  |  |
| --- | --- | --- | --- | --- | --- | --- | --- | --- | --- | --- | --- | --- |
| **Model** | **-2LL** | **# parameters** | ***df*** | **AIC** | **Comparison model** | **Δ χ2** | **Δ *df*** | ***p*** | **A** | **C (95% CI)** | **E** | **s** |
| Fully sat. | 2001.65 | 10 | 838 | 2021.65 | **-** | **-** | **-** | **-** | **-** | **-** | **-** | **-** |
| ACE-s | 2087.62 | 5 | 843 | 2097.62 | Fully sat. | 85.97 | 5 | <.001 | .61 [37; .72] | <.001 [<.001; .37] | .39 [.24; .57] | .03 [-.14; .99] |
| ACE | 2087.97 | 4 | 844 | 2095.97 | ACE-s | 0.34 | 1 | 0.56 | .64 [.38; .71] | <.001 [<.001; .24] | .36 [.29; .44] | - |
| AE-s | 2087.62 | 4 | 844 | 2095.62 | ACE-s | <.001 | 1 | 1.00 | .61 [.43; .72] | - | .39 [.28; .57] | .03 [-.06; .99] |
| AE | 2087.97 | 3 | 845 | 2093.97 | ACE-s | 0.34 | 2 | 0.84 | .64 [.56; .71] | - | .36 [.29; .44] | - |
| CE | 2110.47 | 3 | 845 | 2116.47 | ACE-s | 22.85 | 2 | <.001 | - | .50 [.42; .57] | .50 [.43; .58] | - |
| E | 2219.25 | 2 | 846 | 2223.25 | ACE-s | 131.63 | 3 | <.001 | - | - | 1 | - |

**Table S17**. Bivariate twin model fitting.

| **Wakeups per night** | | |  |  |  |  |  |  |
| --- | --- | --- | --- | --- | --- | --- | --- | --- |
| **Model** | **-2LL** | **# parameters** | ***df*** | **AIC** | **Comparison model** | **Δ χ2** | **Δ *df*** | ***p*** |
| Fully sat. | 3628.29 | 28 | 1542 | 3684.29 | **-** | **-** | **-** | **-** |
| ACE-s | 3649.20 | 13 | 1557 | 3675.20 | Fully sat. | 20.92 | 15 | .14 |
| ACE | 3651.11 | 11 | 1559 | 3673.11 | ACE-s | 1.90 | 2 | .39 |
| AE-s | 3680.67 | 10 | 1560 | 3700.67 | ACE-s | 31.46 | 3 | <.001 |
| AE | 3917.55 | 8 | 1562 | 3933.55 | ACE-s | 268.34 | 5 | <.001 |
| CE | 3738.68 | 8 | 1562 | 3754.68 | ACE-s | 89.48 | 5 | <.001 |
| E | 5025.54 | 5 | 1565 | 5035.54 | ACE-s | 1376.34 | 8 | <.001 |

**Table S18**. Bivariate twin model fitting.

| **Time until settled (daytime)** | | |  |  |  |  |  |  |
| --- | --- | --- | --- | --- | --- | --- | --- | --- |
| **Model** | **-2LL** | **# parameters** | ***df*** | **AIC** | **Comparison model** | **Δ χ2** | **Δ *df*** | ***p*** |
| Fully sat. | 3829.69 | 28 | 1546 | 3885.69 | **-** | **-** | **-** | **-** |
| ACE-s | 3898.11 | 13 | 1561 | 3924.11 | Fully sat. | 68.42 | 15 | <.001 |
| ACE | 3898.70 | 11 | 1563 | 3920.70 | ACE-s | 0.59 | 2 | .74 |
| AE-s | 3899.98 | 10 | 1564 | 3919.98 | ACE-s | 1.87 | 3 | .60 |
| AE | 3925.45 | 8 | 1566 | 3941.45 | ACE-s | 27.34 | 5 | <.001 |
| CE | 3933.25 | 8 | 1566 | 3949.25 | ACE-s | 35.14 | 5 | <.001 |
| E | 4279.66 | 5 | 1569 | 4289.66 | ACE-s | 381.55 | 8 | <.001 |

**Table S19**. Bivariate twin model fitting.

| **Time until settled (evening)** | | |  |  |  |  |  |  |
| --- | --- | --- | --- | --- | --- | --- | --- | --- |
| **Model** | **-2LL** | **# parameters** | ***df*** | **AIC** | **Comparison model** | **Δ χ2** | **Δ *df*** | ***p*** |
| Fully sat. | 3964.43 | 28 | 1541 | 4020.43 | **-** | **-** | **-** | **-** |
| ACE-s | 4005.31 | 13 | 1556 | 4031.31 | Fully sat. | 40.88 | 15 | <.001 |
| ACE | 4007.76 | 11 | 1558 | 4029.76 | ACE-s | 2.45 | 2 | .29 |
| AE-s | 4008.06 | 10 | 1559 | 4028.06 | ACE-s | 2.75 | 3 | .43 |
| AE | 4019.84 | 8 | 1561 | 4035.84 | ACE-s | 14.53 | 5 | .01 |
| CE | 4027.09 | 8 | 1561 | 4043.09 | ACE-s | 21.78 | 5 | <.001 |
| E | 4333.09 | 5 | 1564 | 4343.09 | ACE-s | 327.79 | 8 | <.001 |

**Table S20**. Bivariate twin model fitting.

| **Time until settled (nighttime)** | | |  |  |  |  |  |  |
| --- | --- | --- | --- | --- | --- | --- | --- | --- |
| **Model** | **-2LL** | **# parameters** | ***df*** | **AIC** | **Comparison model** | **Δ χ2** | **Δ *df*** | ***p*** |
| Fully sat. | 3952.56 | 28 | 1554 | 4008.56 | **-** | **-** | **-** | **-** |
| ACE-s | 4012.38 | 13 | 1569 | 4038.38 | Fully sat. | 59.82 | 15 | <.001 |
| ACE | 4013.49 | 11 | 1571 | 4035.49 | ACE-s | 1.11 | 2 | .57 |
| AE-s | 4016.09 | 10 | 1572 | 4036.09 | ACE-s | 3.71 | 3 | .29 |
| AE | 4048.17 | 8 | 1574 | 4064.17 | ACE-s | 35.79 | 5 | <.001 |
| CE | 4028.01 | 8 | 1574 | 4044.01 | ACE-s | 15.63 | 5 | .01 |
| E | 4372.58 | 5 | 1577 | 4382.58 | ACE-s | 360.20 | 8 | <.001 |

**Table S21**. Bivariate twin model fitting.

| **Crying duration (daytime)** | | |  |  |  |  |  |  |
| --- | --- | --- | --- | --- | --- | --- | --- | --- |
| **Model** | **-2LL** | **# parameters** | ***df*** | **AIC** | **Comparison model** | **Δ χ2** | **Δ *df*** | ***p*** |
| Fully sat. | 3692.92 | 28 | 1507 | 3748.92 | **-** | **-** | **-** | **-** |
| ACE-s | 3762.27 | 13 | 1522 | 3788.27 | Fully sat. | 69.35 | 15 | <.001 |
| ACE | 3768.85 | 11 | 1524 | 3790.85 | ACE-s | 6.58 | 2 | .04 |
| AE-s | 3762.27 | 10 | 1525 | 3782.27 | ACE-s | <.001 | 3 | 1.00 |
| AE | 3797.21 | 8 | 1527 | 3813.21 | ACE-s | 34.94 | 5 | <.001 |
| CE | 3793.17 | 8 | 1527 | 3809.17 | ACE-s | 30.90 | 5 | <.001 |
| E | 4228.21 | 5 | 1530 | 4238.21 | ACE-s | 465.94 | 8 | <.001 |

**Table S22**. Bivariate twin model fitting.

| **Crying duration (evening)** | | |  |  |  |  |  |  |
| --- | --- | --- | --- | --- | --- | --- | --- | --- |
| **Model** | **-2LL** | **# parameters** | ***df*** | **AIC** | **Comparison model** | **Δ χ2** | **Δ *df*** | ***p*** |
| Fully sat. | 3740.61 | 28 | 1509 | 3796.61 | **-** | **-** | **-** | **-** |
| ACE-s | 3792.21 | 13 | 1524 | 3818.21 | Fully sat. | 51.60 | 15 | <.001 |
| ACE | 3794.66 | 11 | 1526 | 3816.66 | ACE-s | 2.45 | 2 | .29 |
| AE-s | 3792.43 | 10 | 1527 | 3812.43 | ACE-s | 0.22 | 3 | .97 |
| AE | 3805.71 | 8 | 1529 | 3821.71 | ACE-s | 13.50 | 5 | .02 |
| CE | 3850.06 | 8 | 1529 | 3866.06 | ACE-s | 57.85 | 5 | <.001 |
| E | 4261.64 | 5 | 1532 | 4271.64 | ACE-s | 469.43 | 8 | <.001 |

**Table S23**. Bivariate twin model fitting.

| **Crying duration (nighttime)** | | |  |  |  |  |  |  |
| --- | --- | --- | --- | --- | --- | --- | --- | --- |
| **Model** | **-2LL** | **# parameters** | ***df*** | **AIC** | **Comparison model** | **Δ χ2** | **Δ *df*** | ***p*** |
| Fully sat. | 3716.44 | 28 | 1522 | 3772.44 | **-** | **-** | **-** | **-** |
| ACE-s | 3903.67 | 13 | 1537 | 3929.67 | Fully sat. | 187.22 | 15 | <.001 |
| ACE | 3907.71 | 11 | 1539 | 3929.71 | ACE-s | 4.04 | 2 | .13 |
| AE-s | 3903.67 | 10 | 1540 | 3923.67 | ACE-s | <.001 | 3 | 1.00 |
| AE | 3916.47 | 8 | 1542 | 3932.47 | ACE-s | 12.81 | 5 | .03 |
| CE | 3932.78 | 8 | 1542 | 3948.78 | ACE-s | 29.11 | 5 | <.001 |
| E | 4135.02 | 5 | 1545 | 4145.02 | ACE-s | 231.35 | 8 | <.001 |

**Table S24**. GEE analyses of polygenic scores and sleep/settle behaviors.

|  | **Standardized β (p-value)** | | | | | | | | | | | | | |
| --- | --- | --- | --- | --- | --- | --- | --- | --- | --- | --- | --- | --- | --- | --- |
|  | **2 months** | | | | | | | **5 months** | | | | | | |
|  | Nighttime awakenings | Time until settled (daytime) | Time until settled (evening) | Time until settled (nighttime) | Crying duration (daytime) | Crying duration (evening) | Crying duration (nighttime) | Nighttime awakenings | Time until settled (daytime) | Time until settled (evening) | Time until settled (nighttime) | Crying duration (daytime) | Crying duration (evening) | Crying duration (nighttime) |
| **ADHD** | 0.07 (.12) | -0.03 (.64) | 0.01 (.89) | 0.03 (.50) | 0.11 (.01) | 0.09 (.04) | 0.04 (.35) | 0.04 (.34) | 0.06 (.12) | 0.06 (.21) | 0.10 (.02) | 0.07 (.06) | 0.09 (.03) | 0.09 (.08) |
| **Autism** | -0.01 (.86) | -0.01 (.81) | 0.03 (.48) | -0.04 (.28) | 0.08 (.08) | 0.16 (.002)* | -0.08 (.16) | 0.06 (.17) | 0.01 (.87) | -0.02 (.63) | -0.04 (.24) | -0.01 (.84) | <0.01 (.92) | -0.08 (.06) |
| **Anxiety** | 0.06 (.19) | <0.01 (.96) | 0.04 (.32) | 0.02 (.66) | -0.06 (.14) | -0.02 (68) | <0.01 (.95) | -0.01 (.80) | 0.01 (.74) | 0.03 (.43) | 0.09 (.02) | -0.10 (.02) | 0.01 (.81) | -0.05 (.15) |
| **Depression** | 0.07 (.21) | -0.01 (.80) | -0.01 (.83) | -0.02 (.59) | 0.02 (.70) | -0.01 (.91) | -0.03 (.60) | 0.03 (.47) | <-0.01 (.91) | -0.02 (.62) | -0.05 (.15) | -0.03 (.50) | -0.05 (.18) | 0.04 (0.27) |
| **Circadian rhythm** | -0.11 (.03) | 0.01 (.81) | <-0.01 (.91) | -0.02 (.76) | -0.09 (.03) | -0.04 (.29) | -0.02 (.62) | -0.07 (.09) | 0.02 (.52) | -0.02 (.68) | -0.05 (.29) | -0.02 (.65) | 0.03 (.56) | -0.03 (.57) |
| **Insomnia** | 0.01 (.77) | -0.08 (.07) | 0.06 (.17) | 0.06 (.13) | 0.02 (.81) | -0.01 (.81) | -0.01 (.77) | 0.04 (.39) | 0.02 (.64) | <-0.01 (.93) | -0.02 (.60) | 0.02 (.68) | -0.03 (.33) | 0.01 (.86) |
| **Short sleep** | 0.02 (.75) | -0.06 (.28) | 0.04 (.32) | 0.01 (.75) | -0.03 (.68) | -0.02 (.56) | -0.06 (.09) | 0.07 (.13) | -0.01 (.81) | -0.07 (.11) | -0.02 (.71) | -0.06 (.19) | -0.07 (.08) | -0.03 (.50) |
| **Long sleep** | 0.04 (.40) | 0.01 (.75) | <0.01 (.96) | -0.05 (.12) | 0.02 (.68) | 0.03 (.50) | -0.01 (.70) | 0.03 (.58) | -0.04 (.33) | <-0.01 (.91) | 0.03 (.39) | 0.02 (.72) | -0.01 (.81) | 0.02 (.67) |

*p<.004

**References**

Austin-Zimmerman, I., Levey, D. F., Giannakopoulou, O., Deak, J. D., Galimberti, M., Adhikari, K., Zhou, H., Denaxas, S., Irizar, H., Kuchenbaecker, K., McQuillin, A., Concato, J., Buysse, D. J., Gaziano, J. M., Gottlieb, D. J., Polimanti, R., Stein, M. B., Bramon, E., & Gelernter, J. (2023). Genome-wide association studies and cross-population meta-analyses investigating short and long sleep duration. *Nature Communications 2023 14:1*, *14*(1), 1–15. https://doi.org/10.1038/s41467-023-41249-y

Chang, C. C., Chow, C. C., Tellier, L. C. A. M., Vattikuti, S., Purcell, S. M., & Lee, J. J. (2015). Second-generation PLINK: Rising to the challenge of larger and richer datasets. *GigaScience*, *4*(1), 7. https://doi.org/10.1186/S13742-015-0047-8/2707533

Delaneau, O., Zagury, J. F., Robinson, M. R., Marchini, J. L., & Dermitzakis, E. T. (2019). Accurate, scalable and integrative haplotype estimation. *Nature Communications 2019 10:1*, *10*(1), 1–10. https://doi.org/10.1038/s41467-019-13225-y

Demontis, D., Walters, G. B., Athanasiadis, G., Walters, R., Therrien, K., Nielsen, T. T., Farajzadeh, L., Voloudakis, G., Bendl, J., Zeng, B., Zhang, W., Grove, J., Als, T. D., Duan, J., Satterstrom, F. K., Bybjerg-Grauholm, J., Bækved-Hansen, M., Gudmundsson, O. O., Magnusson, S. H., … Børglum, A. D. (2023). Genome-wide analyses of ADHD identify 27 risk loci, refine the genetic architecture and implicate several cognitive domains. *Nature Genetics 2023 55:2*, *55*(2), 198–208. https://doi.org/10.1038/s41588-022-01285-8

Ge, T., Chen, C. Y., Ni, Y., Feng, Y. C. A., & Smoller, J. W. (2019). Polygenic prediction via Bayesian regression and continuous shrinkage priors. *Nature Communications 2019 10:1*, *10*(1), 1–10. https://doi.org/10.1038/s41467-019-09718-5

Grove, J., Ripke, S., Als, T. D., Mattheisen, M., Walters, R. K., Won, H., Pallesen, J., Agerbo, E., Andreassen, O. A., Anney, R., Awashti, S., Belliveau, R., Bettella, F., Buxbaum, J. D., Bybjerg-Grauholm, J., Bækvad-Hansen, M., Cerrato, F., Chambert, K., Christensen, J. H., … Børglum, A. D. (2019). Identification of common genetic risk variants for autism spectrum disorder. *Nature Genetics*, *51*(3), 431. https://doi.org/10.1038/S41588-019-0344-8

Howard, D. M., Adams, M. J., Clarke, T. K., Hafferty, J. D., Gibson, J., Shirali, M., Coleman, J. R. I., Hagenaars, S. P., Ward, J., Wigmore, E. M., Alloza, C., Shen, X., Barbu, M. C., Xu, E. Y., Whalley, H. C., Marioni, R. E., Porteous, D. J., Davies, G., Deary, I. J., … McIntosh, A. M. (2019). Genome-wide meta-analysis of depression identifies 102 independent variants and highlights the importance of the prefrontal brain regions. *Nature Neuroscience 2019 22:3*, *22*(3), 343–352. https://doi.org/10.1038/s41593-018-0326-7

Jones, S. E., Lane, J. M., Wood, A. R., van Hees, V. T., Tyrrell, J., Beaumont, R. N., Jeffries, A. R., Dashti, H. S., Hillsdon, M., Ruth, K. S., Tuke, M. A., Yaghootkar, H., Sharp, S. A., Jie, Y., Thompson, W. D., Harrison, J. W., Dawes, A., Byrne, E. M., Tiemeier, H., … Weedon, M. N. (2019). Genome-wide association analyses of chronotype in 697,828 individuals provides insights into circadian rhythms. *Nature Communications 2019 10:1*, *10*(1), 1–11. https://doi.org/10.1038/s41467-018-08259-7

Marees, A. T., de Kluiver, H., Stringer, S., Vorspan, F., Curis, E., Marie-Claire, C., & Derks, E. M. (2018). A tutorial on conducting genome-wide association studies: Quality control and statistical analysis. *International Journal of Methods in Psychiatric Research*, *27*(2). https://doi.org/10.1002/MPR.1608

Otowa, T., Hek, K., Lee, M., Byrne, E. M., Mirza, S. S., Nivard, M. G., Bigdeli, T., Aggen, S. H., Adkins, D., Wolen, A., Fanous, A., Keller, M. C., Castelao, E., Kutalik, Z., Der Auwera, S. V., Homuth, G., Nauck, M., Teumer, A., Milaneschi, Y., … Hettema, J. M. (2016). Meta-analysis of genome-wide association studies of anxiety disorders. *Molecular Psychiatry 2016 21:10*, *21*(10), 1391–1399. https://doi.org/10.1038/mp.2015.197

Rubinacci, S., Delaneau, O., & Marchini, J. (2020). Genotype imputation using the Positional Burrows Wheeler Transform. *PLOS Genetics*, *16*(11), e1009049. <https://doi.org/10.1371/JOURNAL.PGEN.1009049>

Watanabe, K., Jansen, P.R., Savage, J.E. , Nandakumar, P., Wang, X., … Posthuma, D. (2022) Genome-wide meta-analysis of insomnia prioritizes genes associated with metabolic and psychiatric pathways. *Nat Genet, 54*, 1125–1132. doi: 10.1038/s41588-022-01124-w

Yengo, L., Vedantam, S., Marouli, E., Sidorenko, J., Bartell, E., Sakaue, S., Graff, M., Eliasen, A. U., Jiang, Y., Raghavan, S., Miao, J., Arias, J. D., Graham, S. E., Mukamel, R. E., Spracklen, C. N., Yin, X., Chen, S. H., Ferreira, T., Highland, H. H., … Hirschhorn, J. N. (2022). A saturated map of common genetic variants associated with human height. *Nature*, *610*(7933), 704. https://doi.org/10.1038/S41586-022-05275-Y
